# The Cost Effectiveness of a Tailored, Web-Based Care Program to Enhance Postoperative Recovery in Gynecologic Patients in Comparison With Usual Care: Protocol of a Stepped Wedge Cluster Randomized Controlled Trial

**DOI:** 10.2196/resprot.3236

**Published:** 2014-06-18

**Authors:** Esther VA Bouwsma, Johannes R Anema, Antonie Vonk Noordegraaf, Dirk L Knol, Judith E Bosmans, Steven E Schraffordt Koops, Paul JM van Kesteren, W Marchien van Baal, Jos P Lips, Mark H Emanuel, Petrus C Scholten, Alexander Mozes, Albert H Adriaanse, Hans AM Brölmann, Judith AF Huirne

**Affiliations:** ^1^Department of Obstetrics and GynecologyVU University Medical CenterAmsterdamNetherlands; ^2^Department of Public and Occupational HealthVU University Medical CenterAmsterdamNetherlands; ^3^EMGO Institute for Health and Care ResearchAmsterdamNetherlands; ^4^Department of Epidemiology and StatisticsVU University Medical CenterAmsterdamNetherlands; ^5^Department of Health SciencesFaculty of Earth and Life SciencesVU UniversityAmsterdamNetherlands; ^6^Department of Obstetrics and GynecologyMeander Medical CenterAmersfoortNetherlands; ^7^Department of Obstetrics and GynecologyOnze Lieve Vrouwe GasthuisAmsterdamNetherlands; ^8^Department of Obstetrics and GynecologyFlevo HospitalAlmereNetherlands; ^9^Department of Obstetrics and GynecologyKennemer GasthuisHaarlemNetherlands; ^10^Department of Obstetrics and GynaecologySpaarne HospitalHoofddorpNetherlands; ^11^Department of Obstetrics and GynaecologyDiakonessenhuisUtrechtNetherlands; ^12^Department of Obstetrics and GynecologyAmstelland HospitalAmstelveenNetherlands; ^13^Department of Obstetrics and GynecologyMedical Center AlkmaarAlkmaarNetherlands

**Keywords:** gynecology, Internet, telemedicine, convalescence, return to work, economic evaluation

## Abstract

**Background:**

The length of recovery after benign gynecological surgery and return to work frequently exceeds the period that is recommended or expected by specialists. A prolonged recovery is associated with a poorer quality of life. In addition, costs due to prolonged sick leave following gynecological surgery cause a significant financial burden on society.

**Objective:**

The objective of our study was to present the protocol of a stepped wedge cluster randomized controlled trial to evaluate the cost effectiveness of a new care program for patients undergoing hysterectomy and/or adnexal surgery for benign disease, compared to the usual care.

**Methods:**

The care program under study, designed to improve convalescence and to prevent delayed return to work, targets two levels. At the hospital level, guidelines will be distributed among clinical staff in order to stimulate evidence-based patient education. At the patient level, additional perioperative guidance is provided by means of an eHealth intervention, equipping patients with tailored convalescence advice, and an occupational intervention is available for those patients at risk of prolonged sick leave. Due to the stepped wedge design of the trial, the care program will be sequentially rolled out among the 9 participating hospitals, from which the patients are recruited. Eligible for this study are employed women, 18-65 years of age, who are scheduled for hysterectomy and/or laparoscopic adnexal surgery. The primary outcome is full sustainable return to work. The secondary outcomes include general recovery, quality of life, self-efficacy, coping, and pain. The data will be collected by means of self-reported electronic questionnaires before surgery and at 2, 6, 12, 26, and 52 weeks after surgery. Sick leave and cost data are measured by monthly sick leave calendars, and cost diaries during the 12 month follow-up period. The economic evaluation will be performed from the societal perspective. All statistical analyses will be conducted according to the intention-to-treat principle.

**Results:**

The enrollment of the patients started October 2011. The follow-up period will be completed in August 2014. Data cleaning or analysis has not begun as of this article’s submission.

**Conclusions:**

We hypothesize the care program to be effective by means of improving convalescence and reducing costs associated with productivity losses following gynecological surgery. The results of this study will enable health care policy makers to decide about future implementation of this care program on a broad scale.

**Trial Registration:**

Netherlands Trial Register: NTR2933; http://www.trialregister.nl/trialreg/admin/rctview.asp?TC=2933 (Archived by WebCite at http://www.webcitation.org/6Q7exPG84).

## Introduction

### Early Discharge From the Hospital

In the last two decades, the hospital stay following surgical procedures has been shortened drastically, due to recovery-enhancing strategies such as the use of minimally invasive techniques and the implementation of fast-track programs [1-4]. The advantages of early postoperative discharge include increased patient satisfaction, low hospital-acquired infection rates, and reduced hospitalization costs [5]. However, a major disadvantage of minimizing the length of a hospitalization is that patient contact becomes very brief, which is often at the expense of time spent on patient education. Ironically, the lack of detailed convalescence instructions at the time of discharge increases the risk of an unnecessary prolonged recovery [6-11]. Therefore, as long as the organization of perioperative care has not fully anticipated the transition of postoperative recovery to the home setting, early discharge does not necessarily translate into accelerated recovery and earlier resumption of (work) activities [12-14].

In gynecology, the postoperative convalescence after discharge from the hospital has not received much attention in research and practice. Yet, there is considerable evidence that the length of recovery time after a gynecological surgery systematically exceeds the period considered as appropriate by specialists [5,10,12-17]. In a prospective study performed by our own study group among 148 patients receiving gynecological surgery for a benign disease, median time to return to work (RTW) exceeded the recommended sick leave of 6 weeks by approximately 3 weeks. The median time to RTW following an intermediate surgery (eg, laparoscopic or vaginal hysterectomy) was 60 days (interquartile range, IQR 56-135) and following a major surgery (eg, abdominal hysterectomy) 69 days (IQR 56-135) [10].

### Prolonged Recovery at Home

An unnecessary prolonged recovery is associated with poorer quality of life [18,19]. In addition, work related problems have also been associated with an increase in health care consumption [20]. Furthermore, taken into account that about 14,000 hysterectomies are performed annually in the Netherlands alone [21], the financial burden on society due to delayed convalescence after a gynecological surgery is substantial.

In order to reduce unnecessary delayed recovery, and concurrently decrease costs associated with prolonged sick leave and increased health care utilization following gynecological surgery, our research group started working on an innovative strategy to optimize perioperative care in 2008. Since the beginning of the project several goals were achieved, starting with the development of detailed convalescence recommendations following 4 types of benign gynecological surgery, using a modified Delphi method [22]. Simultaneously, a multidisciplinary care program was developed [23,24] consisting of an interactive eHealth intervention and—for those patients at risk of prolonged sick leave—an occupational intervention. The care program provides guidance to patients from the moment the surgery is planned, until the full resumption of all activities—including return to work—and encourages patients to take an active role in their own recovery. The care program was subject to an effect evaluation as well as a process evaluation in 2010 [25]. While the effectiveness study among 215 patients showed a positive effect on the outcomes: (1) RTW, (2) quality of life, and (3) perceived pain [26], the process evaluation showed some room for improvement [27].

Besides evaluating the effectiveness of a study, it is of equal importance to conduct an economic evaluation, especially considering the high economic burden of extended time to convalescence after a gynecologic surgery. The economic evaluations are necessary to gain insight into the costs of an intervention in relation to its effects. Health care policy makers can use these results to decide how resources should optimally be allocated to maximize health or welfare [28].

Therefore, the primary objective of the current study is to conduct an economic evaluation of the care program compared to the usual care. This economic evaluation will be conducted alongside a randomized trial, as the intervention concerns a further developed version of the care program, which has not yet been subject to an effect evaluation. In addition, this construction enables the systematic collection of relevant effect and cost data under “real life” conditions. As the intervention care program targets two levels (the hospital level and the patient level), a cluster design was chosen in order to prevent contamination between the study arms. The primary outcome duration until full sustainable RTW will be assessed on the level of the individual participant. On the level of the participating hospitals, we will investigate to what extent the guidelines on convalescence recommendations are adopted, and how future implementation of the guidelines and care program can be facilitated.

## Methods

### The Standard Protocol Items

The Standard Protocol Items, Recommendations for Interventional Trials statement [29], and CONsolidated Standards Of Reporting Trials (CONSORT) statement [30,31], were used in order to describe the design of this study. In addition, we used the extension to cluster randomized trials [32] and the CONSORT eHealth checklist [33].

### Ethical Issues

The Institutional Review Boards of all participating hospitals approved this study protocol. Informed consent was obtained from all of the patients.

### Trial Design

**Figure 1:**
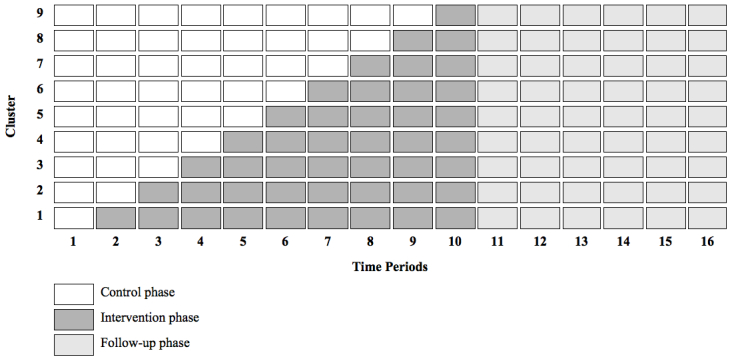
Trial design.

This trial is designed as a cluster, randomized controlled, stepped wedge trial, which involves a sequential rollout of the intervention in the participating clusters over several time periods. In our study, clusters are the departments of obstetrics and gynecology in nine different hospitals in the Netherlands. Each time period (TP) takes 2 months. At the start of the trial (TP_1_), all of the patients scheduled for a surgery in all of the participating hospitals receive usual care (control phase). After two months (TP_2_), the intervention is implemented in the first cluster, and from now on the patients scheduled for a surgery in this hospital will receive the intervention program, while in all of the other hospitals the patients still receive usual care. The patients in cluster 2 who underwent surgery during TP_1_ remain in the control group until they finish the 12 month follow-up. During TP_3_, the intervention program continues in cluster 1, and the intervention is implemented in cluster 2 as well, resulting in the deliverance of the intervention program to the patients in clusters 1 and 2 that will undergo surgery from this point onward, while patients in clusters 3 to 9 serve as the control group. At the beginning of TP_4_, cluster 3 starts with the intervention, etc. This is repeated until the intervention is implemented in all clusters (TP_10_). Figure 1 illustrates the study design.

A cluster design was chosen to minimize the risk of contamination, as our intervention targets both health care providers and patients. A stepped wedge approach was employed because of the unique feature of an unidirectional crossover, preventing the intervention to be withdrawn from the hospital during the trial [34-36]. Because there is substantial evidence from our previous trial that the care program under study will be effective, this is particularly convenient, as hospitals will be able to keep using the intervention after the trial. Moreover, it enables us to study the implementation process carefully, giving valuable insight into barriers and facilitators for future broader implementation.

### Selection of Clusters

The clusters in this trial consist of nine hospitals in the surroundings of Amsterdam, the capital of the Netherlands. The hospitals were eligible if they performed at least 100 hysterectomies or laparoscopic adnexal surgeries yearly, and were located within 50 km of the Vrije Universiteit Medical Center (VUmc). The research team enrolled the clusters before the start of the trial. In an attempt to select a heterogeneous sample of hospitals, we included 1 university hospital, 7 teaching hospitals, and 1 nonteaching hospital.

### Study Population

The eligible participants for this study are women 18-65 years of age, employed for at least 8 hours per week (salary employed, self employed, or voluntary work), and scheduled for a surgery for a benign gynecological disease in one of the nine participating hospitals. The types of surgeries that are included are: (1) total abdominal hysterectomy (TAH), (2) vaginal hysterectomy (VH), (3) total laparoscopic hysterectomy or laparoscopic assisted vaginal hysterectomy (TLH), or (4) laparoscopic adnexal surgery (LAS). The factors that are possibly complicating the postoperative course (eg, severe comorbidity, malignancy, pregnancy), the factors that are interfering with the eHealth intervention (computer or Internet illiteracy), or with the occupational intervention (conflict with employer, prolonged sick leave, or disability) serve as the exclusion criteria. Table 1 lists an overview of all eligibility criteria.

**Table 1:** Eligibility criteria.

Inclusion criteria		Exclusion criteria
Women scheduled for:		(Suspicion of) malignancy
	Laparoscopic adnexal surgery	(Ectopic) pregnancy
	Total laparoscopic hysterectomy	Deep infiltrating endometriosis
	Vaginal hysterectomy	Concomitant health problems affecting daily activities
	Total abdominal hysterectomy	Psychiatric disorders affecting daily activities
18-65 years of age		Legal conflict with employer
Employed ≥ 8 hours/week		Being sick listed >4 weeks, or when reason of sick leave is related to gynecological surgery > 2 months
		Inability to understand or complete Dutch questionnaires
		Computer or Internet illiteracy

### Recruitment of Patients

The recruitment of patients will take place in all participating hospitals. When the patients are scheduled for a hysterectomy or laparoscopic adnexal surgery, they will receive a letter about the study on behalf of their gynecologist. The letter includes detailed information about the trial. In addition, it is explained that someone from the research team will make contact by telephone after one week to evaluate the patients’ willingness to participate and answer questions if necessary. If the patient does not wish to be contacted, she can return an included reply card, or send an email to a specified email address.

When contact is made and the patient is willing to participate, eligibility is assessed. The eligible patients are then requested to return a signed informed consent, which is also attached to the information letter. The participants will not receive any financial or nonfinancial incentives.

### Randomization

The randomization takes place at the level of the clusters and determines the order in which the intervention program is implemented in the participating hospitals. The randomization will be performed by a statistician using a computer generated list of random numbers.

The patients are informed about the allocation of treatment by the research team after the patient’s informed consent and the completion of the first questionnaire before surgery. As the treatment allocation depends on the scheduled date of the surgery, and the implementation phase of the hospital in which they are being operated, it is predetermined for each participant, potentially causing selection bias. To minimize the risk of selection bias, the participants will not be informed about the study design, and will be counseled as if they have equal chances between receiving the usual care or the intervention program. For this reason, counseling will be done by the research team, rather than by their own physician, who might be, for example, more willing to include patients during the intervention phase than during the control phase. Moreover, physicians will be blinded to the randomization schedule, and will only be informed about the start of the intervention phase approximately one month before the actual implementation. Once the intervention phase has started, the importance of not communicating this information with the potential patients will be emphasized.

### Interventions

#### Usual Care

Before the implementation of the intervention program, the participants receive the usual perioperative care as provided in the hospital in which they are scheduled for surgery. Although considerable variation exists in the Netherlands, in most cases patients get verbal (general) instructions at discharge by a nurse and/or physician, often followed—but not necessarily—by a letter or brochure. In general, an outpatient postoperative consultation is scheduled 4 to 6 weeks following the surgery. Between discharge and the postoperative consultation, medical care is only initiated by the patient, who can consult her general physician (GP) or gynecologist, if necessary. Employed workers who have not resumed work within 6 weeks after the surgical procedure will be invited for a consultation with their occupational physician (OP), as required by law in the Netherlands.

#### Intervention

The systematic development of the care program using the principles of Intervention Mapping is described in more detail elsewhere [23]. Both theory and practice were combined, and all stakeholders were involved in the process. The engagement of the patients was prompted through focus groups [24]. The Attitude, Social influence, and Self-efficacy model was used as a theoretical framework for determinants of behavior regarding return to work [37,38].

**Figure 2:**
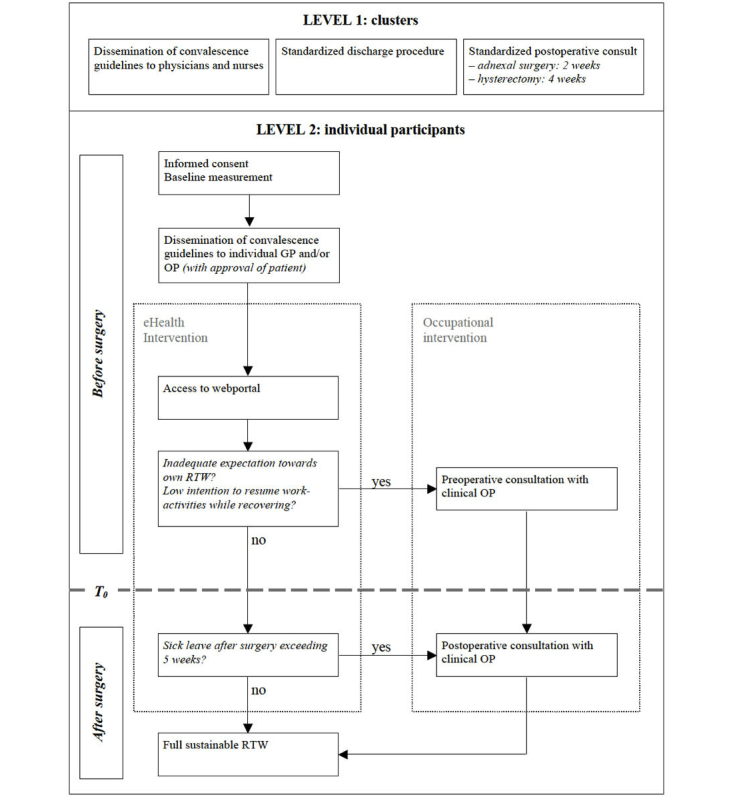
Overview of the care program. GP=general physician; OP= occupational physician; RTW=return to work.

The care program targets two levels, which are described below. Figure 2 shows an overview of the intervention care program.

#### Cluster Level

At the cluster level, the intervention care program aims to structure and stimulate evidence-based perioperative care. Approximately two months before a cluster shifts from the control to the intervention phase, the principle researcher will approach the head of the department to arrange logistics. A minimum of two meetings is planned one or two weeks before the actual implementation with physicians and nurses to provide and explain the new convalescence recommendations that should be communicated to the patients. In addition, all health professionals involved in the clinical care receive a pocket card on which these recommendations are summarized for quick reference. The residents involved in the discharge communication are instructed to explain the convalescence recommendations to their patients before they are discharged. Visual reminders in the patient records will help the residents do so. With the secretary of the department, a strategy is developed to prompt the standard postoperative consultation at 4 weeks following a hysterectomy, and 2 weeks following adnexal surgery. During the trial, newsletters will be spread regularly to reinforce the different aspects of the intervention care program.

#### Patient Level

##### Individual Tailored Guidance

At the patient level, the care program aims to provide individual tailored guidance to patients from the moment the surgery is planned until the full resumption of all activities. It consists of two steps: (1) access to an interactive eHealth intervention for all patients, and (2) an additional occupational intervention for those patients at risk for prolonged sick leave.

##### eHealth Intervention

**Figure 3:**
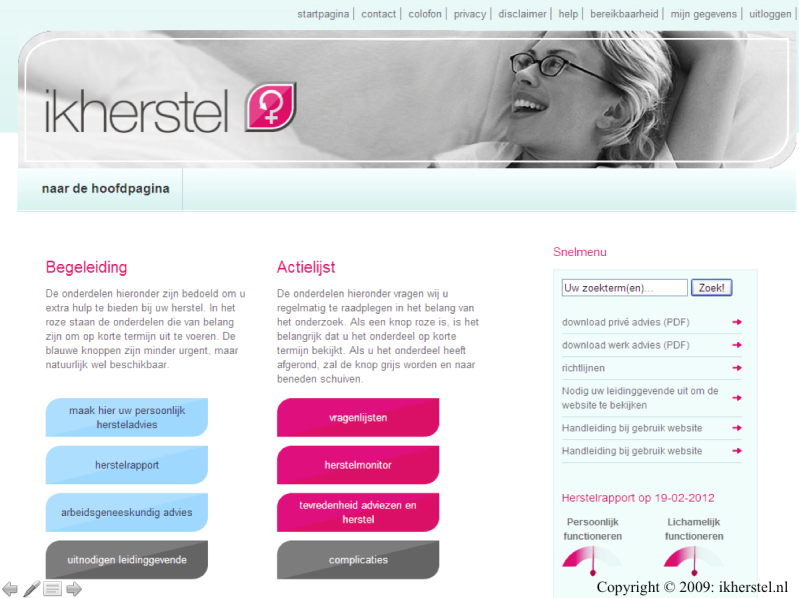
Screenshot of ikherstel.

The patient webportal (Figure 3, [39] aims at empowering its users and improving communication between patients and their employers, as well as improving the communication between the involved health care professionals during the perioperative period. Access to the webportal will be given to the patients approximately 2 to 4 weeks prior to surgery by the research team, by providing a username and temporary password. The instructions are given by email, and it is explained that if patients require assistance, they can contact the research team by phone or email. If patients fail to log in, an automatic reminder is sent to them one week before their surgery to remind them about the webportal and its functionalities. User authentication will make it possible to analyze website activity for each individual participant (visit duration, number of sessions, number and details of pages visited).

The most important tool of the webportal is the possibility to generate a tailored convalescence plan. In the instruction email, patients are encouraged to generate such a plan at least once, preferably before surgery. Having access to detailed convalescence advice will enable the patients to develop realistic expectations about their own recovery, and plan the resumption of their activities and work reintegration accordingly. Moreover, a tailored convalescence plan will help the patients gain insight into potential recovery problems and find solutions at an early stage, preferably before surgery. Because the convalescence plan is composed before surgery, gynecologists are asked to approve the plan electronically on the first postoperative day. In the case of an uncomplicated procedure, the plan is turned into a definite convalescence plan, and the patients are instructed to follow the recommendations in it. In the case of a converted procedure, the plan is adjusted to the type of surgery that was actually performed. In the event of severe complications, the gynecologist can choose not to approve the convalescence plan, and the patients then receive a message that the convalescence plan is not valid anymore, and that they should follow up with the specific instructions given to them at discharge. With the consent of the patient, the approved convalescence plan is also disclosed to the GP and/or OP of the patient. This last feature was added since the prior evaluation of the webportal, and was developed to facilitate the involvement of other health care professionals during the perioperative period in order to stimulate a multidisciplinary approach. In addition, the webportal was equipped with a tool that enables the patients to generate a recovery report, a graphic presentation of their own recovery, allowing them to track their progress.

During the trial, the content of the website will be frozen, except from the dynamic component (forum). Table 2 summarizes the most important tools of the eHealth intervention. Screenshots of the webportal are included as a Multimedia Appendix (see Multimedia Appendix 1).

**Table 2:** Content of the eHealth intervention.

Tool	Description
Personalized convalescence plan^a^	The tool allows patients to generate detailed tailored instructions on the resumption of activities after the surgery, allowing preoperative planning of (work) activities. The convalescence plan is approved electronically on the first postoperative day by the surgeon who performed the surgery, resulting in a definitive convalescence plan. With the consent of the patient, the approved convalescence plan is shared with GP and/or OP.^a^
Recovery monitor + recovery report^a^	The tool makes an inventory of the resumption of activities at 2, 4, 7, 14, 28, 56, and 84 days after surgery. Results are graphically displayed in a recovery report, allowing the patient to track their progress.^a^ In case the patients fall behind, an alerting system advises them to contact a specific health care professional, depending on the underlying problem.
Invitation of employer	The tool allows patients to invite an employer to an anonymous section of the webportal to stimulate a dialogue. The development of a reintegration plan preoperatively will help them gain insight into potential RTW problems.
Video	There is a 9-minute film illustrating the common pitfalls during the postoperative period.
Knowledge	There are several tools to find additional information, such as an extended list with answers to frequently asked questions, a glossary, and links to other useful websites.
Forum	The tool allows the patients to interact (privately or publicly) with other patients.

^a^Tools that were modified since the last evaluation of the webportal

##### Occupational Intervention

The occupational intervention is developed to provide additional guidance to those patients at risk for prolonged sick leave. The occupational intervention will be delivered by a group of six independent OPs, who will be trained as RTW coordinators before the start of the trial. There are two types of consultations: (1) a preoperative, and (2) a postoperative consultation. All consultations will be delivered by telephone, unless the OP and the patient decide together otherwise.

The patients who have an inadequate expectation about their own recovery (longer than 3 weeks for LAS, longer than 6 weeks for VH/TLH, or longer than 8 weeks for TAH), or have a low intention to resume work activities while still recovering, are offered a preoperative consultation, as expectations about RTW and intention to resume work have been identified as two predictors for RTW in recent studies [10,40,41]. During the preoperative consultation, the OP explains the importance of a prosperous recovery in terms of improving quality of life and preventing long term sickness. In addition, the OP tries to identify and—if necessary—alter attitudes and (irrational) beliefs about recovery.

The patients who exceed 5 weeks of sick leave receive a postoperative consultation, during which, the OP assesses the underlying mechanism for the delayed recovery. The OP gives advice to improve the reintegration process. Moreover, as a RTW coordinator, the OP has an excellent position to communicate with the patient’s gynecologist, GP, OP, and employer, if necessary, and of course, with the consent of the patient, stimulating an integrated care approach. In addition, the OP has the possibility to initiate a participatory workplace intervention, aimed at finding consensus between the patient and her employer concerning solutions for identified obstacles for RTW with the help of an occupational therapist (OT) [42,43].

The occupational intervention described above differs from the intervention as delivered during the first trial, due to the insight gained during the process evaluation. Originally, contact with the clinical OP took place in the 10^th^or 11^th^week, however, this turned out to be too late in order to be able to alter attitudes and beliefs, and influence the development of a solid RTW plan. Therefore, in the current trial, contact will be made much earlier, at 5 weeks, and on indication already before surgery. In addition, the patients will receive the details of the postoperative appointments before surgery in order to prepare them that the occupational intervention is part of the care program they receive, as in the prior trial, almost half of the patients declined additional occupational care. In the case of full RTW, the postoperative appointment will be cancelled.

### Outcomes

#### Effect Measures

The effects of the intervention will be assessed on the level of the patient. The primary outcome of the study is the sick leave duration until full sustainable RTW, defined as the duration of the sick leave in calendar days from the day of surgery until full RTW, in their own work or other work with equal earnings, for at least 4 weeks without (partial or full) recurrence [44]. The recurrence of sick leave due to the gynecologic surgery within the four week period after initial full RTW will be added to the preceding period of the sick leave. The RTW will be assessed by a monthly electronic sick leave calendar.

Secondary outcomes that will be assessed are:

Recovery, measured by the Recovery Index-10 (RI-10) a validated recovery-specific questionnaire [45];
Self-reported quality of life, assessed by the Dutch versions of the EuroQol-5D (EQ-5D) [46] and the Short-Form Health Survey (SF-36) [47,48];
Duration of sick leave until first RTW, and total duration of sick leave due to the gynecological surgery for the entire follow-up period, both measured by the monthly sick leave calendars;
Self-efficacy, assessed by the Dutch adaptation of the General Self-Efficacy Scale (GSES) [49];
Coping, assessed by the Pearlin Mastery Scale (PMS) [50];
Pain intensity, measured by the Von Korff questionnaire (VAS) [51]; and
(Post) operative complications both assessed through self-report and by the review of surgical reports. Complications include: (1) enlargement of the wound (≥ 8cm), (2) unintended injury to other structures (eg, bowel, bladder, ureter), (3) unexpected blood loss requiring transfusion, (4) prolonged hospital stay, (5) readmission within 72 hours (overnight), (6) repeat surgery within 2 weeks, and (7) postoperative infection requiring antibiotics.

#### Prognostic Factors

Before surgery, data about potential prognostic factors will be collected. In case of coincidental and meaningful differences, analyses will be adjusted for the following characteristics: (1) sociodemographic data such as age, education level, and ethnicity; (2) personal factors such as expectation, motivation, and intention toward RTW, duration of sick leave in the past 3 months; and (3) work related factors such as physical workload and potential work related psychosocial factors, assessed by the Dutch Musculoskeletal Questionnaire (DMQ) [52] and the Job Content Questionnaire (JCQ) [53].

In case of an unequal distribution of severe complications (defined as: wound enlargement with more than 8cm or repeat surgery within 2 weeks), between the two study arms, the analyses will be adjusted for these surgery-related characteristics as well.

#### Cost Measures

The costs will be measured from a societal perspective and consist of: (1) costs of the intervention, (2) health care utilization, and (3) costs associated with lost productivity. All of the costs will be converted to the year 2014 using consumer price indices [54]. The discounting of costs will not be necessary because the follow-up period is limited to one year.

The intervention costs are those that are related to implementing and operating the new care program, and will be estimated using a bottom-up approach. The detailed information regarding the quantity and unit prices of the following resources will be collected: (1) training of involved health care professionals (clinical staff, OP, OT), (2) the eHealth intervention (hosting of webportal, administrator time), and (3) the occupational intervention (number and duration of consultations).

The health care utilization will be assessed on a monthly basis using a retrospective electronic questionnaire. Only the health care costs related to the gynecological surgery will be collected and include: (1) surgery and hospitalization; (2) visit is to health care professionals in primary or secondary care and visits to alternative medicine therapists; (3) medication; and (4) home care and informal help. If available, Dutch guideline prices will be used to value health care utilization. If cost guidelines are not available, costs will be estimated using real prices or population-based estimates if available in the literature. The prices of the Royal Dutch Society for Pharmacy will be used to value medication [55].

The costs associated with productivity loss consist of absenteeism and presenteeism costs. The absenteeism will be assessed by monthly sick leave calendars. The human capital approach will be used to calculate the costs of losses to production as a result of sick leave due to the gynecologic surgery (net number of days on sick leave during follow-up, multiplied by the estimated prices of production loss of a worker per day of sick leave). The presenteeism (reduced productivity while at work) will be assessed with two items of the Productivity and Disease Questionnaire [56]. A decline in the amount or quality of work performed due to the gynecologic surgery compared to the level at which the patient normally performs, will be considered as presenteeism. The costs associated with presenteeism will be calculated by multiplying the presenteeism score during follow-up by the estimated price of production loss per day.

#### Process Measures

A process evaluation will be conducted to evaluate the implementation process of the intervention [57]. The assessment of the extent to which the intervention program was applied as intended will provide valuable insight into the facilitators and barriers for future implementation. The process evaluation will take place both on the level of the cluster as well as the patient, and both quantitative and qualitative methods will be used. An automatically generated weblog will enable the analysis of the website activity for each individual participant, giving more insight into which patients used the eHealth intervention, and how it is being used. The appointment system and patient records of the OP will enable us to analyze the number of consultations that have taken place, as well as the reasons for cancellations, and the occurrence of any protocol deviations. By means of an Internet questionnaire at the end of the follow-up period, patient satisfaction, perceived effectiveness, and any usage barriers will be assessed. The principle investigator will continuously collect reasons for exclusion and dropout during the trial. In accordance to the prior process evaluation conducted [27], the following process measures are included: (1) reach, extent to which the intervention reaches the target population; (2) dose delivered, extent to which the intervention is delivered to the target population; (3) dose received, extent to which the participants used the intervention; (4) fidelity, extent to which the intervention was delivered as planned; and (5) attitudes, satisfaction, perceived effectiveness, and usage barriers.

### Cointerventions and Contamination

Cointerventions during the intervention period cannot always be avoided. However, we will be able to determine whether patients received cointerventions by means of the monthly cost diaries. The risk of contamination is reduced by the cluster design of the trial. To assess whether contamination occurred, the patients in both groups are asked about the instructions they received at discharge, which will then be compared to the convalescence recommendations implemented during the intervention phase of the study.

### Data Collection

The surgery is considered T_0_. The data will be collected by means of self-reported electronic questionnaires [58] before surgery and 2 weeks (T_1_), 6 weeks (T_2_), 12 weeks (T_3_), 26 weeks (T_4_), and 52 weeks (T_5_) after surgery. In addition, all of the participants will be requested to fill out a monthly electronic sick leave calendar and cost diary. The patients that are not sick listed, and do not have medical costs during 3 consecutive months, receive a shortened version of the monthly questionnaire. In the case of no response, the patients receive an electronic reminder after 1 and, if necessary, 2 weeks. Every 3 months an attempt will be made to complete missing data regarding RTW, sick leave, and health care usage per email, post, and/or telephone. Table 3 provides an overview of all outcome measures and assessment instruments used in this trial. Not all of the instruments have been validated for Internet use.

**Table 3:** Assessment of study outcomes.

Outcome measures		–		+	+	+	+	+
		± 4 weeks	Surgery (T_0_)	2 weeks (T_1_)	6 weeks (T_2_)	3 months (T_3_)	6 months (T_4_)	12 months (T_5_)
**Primary**								
	Duration of sick leave until full sustainable RTW	Monthly sick leave calendar^a^						
**Secondary**								
	Duration of sick leave until first RTW	Monthly sick leave calendar^a^						
	Total duration of sick leave	Monthly sick leave calendar^a^						
	Recovery (RI-10)	x		x	x	x	x	x
	Quality of life (EQ-5D)	x		x	x	x	x	x
	Quality of life (SF-36)	x				x	x	x
	Self-efficacy (GSES)			x		x		x
	Coping (PMS)			x		x		x
	Pain intensity (VAS)			x	x	x	x	x
	(Post) operative complications			x	x			x^b^
**Prognostic factors**								
	Social demographic variables	x						
	Personal factors	x						
	Work related factors (DMQ, JCQ)	x						
	Type of surgery/complications		x					
**Cost**								
	Care program	Bottom-up approach^c^						
	Health care utilization	Monthly cost diary^a^						
	Productivity loss	Monthly sick leave calendar^a^						
**Process** ^d^								
	Compliance (dose received)	Continuously by weblog						
	Attitudes (satisfaction, perceived effectiveness, usage barriers)					x		x
	Satisfaction Patient Satisfaction with Occupational Health Services Questionnaire					x		x

^a^short version after 3 consecutive months without sick leave or health care usage

^b^review of surgical reports

^c^calculated by research team

^d^only intervention group

### Blinding

The participants, care providers, and researchers cannot be blinded for the allocated treatment. However, analysis of the data by the researcher will be blind, as all of the patients receive their own study code, under which their data is stored in the database. The assessment of the outcomes is measured through self-reported questionnaires.

### Sample Size

We calculated the sample size needed with the method described by Hussey and Hughes [35]. Based on the previous study, we expect a hazard ratio of 1.5 on the primary outcome full sustainable RTW. To achieve a power of 0.8 with a two-tailed alpha of .05, and taking into account a dropout rate of 10%, a total of 212 patients will be needed when using the log-rank test.

With an intracluster correlation of .05, 9 clusters, and 10 time periods, the design effect is calculated to be 2.14 [35]. By multiplying the design effect by the sample size without a correction for a stepped wedge design, a sample size of 454 women is needed. Assuming that all of the hospitals will include the same amount of participants, each hospital should include approximately 50 patients (5 patients per time period per hospital).

### Statistical Analyses

#### Effect Evaluation

All further described analyses will be performed at the patient level, according to the intention-to-treat principle. In addition, for all tests, a two-tailed significance level of *P*≤.05 will be considered statistically significant. The statistical software packages that will be used include SPSS (version 16.0) and STATA (version 11.2).

The baseline characteristics will be summarized using descriptive statistics, and compared between the experimental and control group to verify prognostic comparability. In case of coincidental and meaningful differences, these variables will be used as covariates in the further described models.

For the primary outcome, the duration of sick leave until full sustainable RTW, Cox regression analyses will be used to investigate the intervention effect. Both the crude and adjusted analyses will be performed. In the adjusted analyses, the following variables will be used as covariates: (1) hospital, to adjust for clustering (random gamma effect); (2) type of surgery performed; (3) time period, to adjust for naturally occurring changes over time irrespective of the intervention; and (4) optionally, (time period) x (intervention) interaction term, to adjust for time effects (the longer the care program is implemented, the more effective it might be).

The differences in secondary outcomes will be assessed using generalized linear longitudinal mixed models. All of the available measurements (2 weeks, 6 weeks, 12 weeks, 26 weeks, and 52 weeks) will be used, and the baseline scores will be used as covariates, as well as the hospital and the type of surgery (random effect).

To assess whether protocol deviations caused bias, a per protocol analysis will be performed, and the results will be compared to the intention-to-treat analyses. In addition, several subgroup analyses will be performed. The predefined subgroups will be: (1) hysterectomy (TAH, VH, TLH); (2) minimally invasive hysterectomy (VH, TLH); (3) abdominal hysterectomy only; and (4) laparoscopic adnexal surgery only.

#### Economic Evaluation

Both a cost-effectiveness analysis and a cost-utility analysis will be performed from the societal perspective. The analyses will be performed according to the intention-to-treat principle. The missing cost and effect data will be imputed using multiple imputation [59]. The imputation will include variables that are related to the missing data or the outcome measure, and variables that differ at baseline between the groups. To account for the skewed distribution of costs, predictive mean matching will be used in the multiple imputation. The number of imputed datasets to be created will be determined based on the fraction of missing information [60]. 'All of the datasets will be analyzed separately, and the results of these analyses will be pooled using Rubin's rules [61]. The incremental cost effectiveness ratios (ICERs) will be calculated by dividing the differences in mean total costs between both treatment groups, by the difference in mean effects between both treatment groups. To avoid double counting, the productivity costs due to sick leave will be excluded in the ICER, with sick leave as the effect measure. The incremental cost utility ratio will be calculated by dividing the incremental costs by the difference in the quality adjusted life years between both treatment groups. To account for the typically skewed distribution of costs, bias corrected and accelerated bootstrapping (5000 replications) will be used to estimate the 95% confidence intervals around the mean cost differences, and the uncertainty surrounding the ICERs. The bootstrapped ICERs will be graphically presented in cost effectiveness planes [62]. The cost effectiveness acceptability curves will be estimated to show the probability of the intervention program to be cost effective in comparison with the usual care for a range of different ceiling ratios, thereby showing decision uncertainty [63]. To assess the robustness of results, several secondary economic analyses will be performed: (1) complete case analysis, (2) per protocol analysis, (3) analysis with costs calculated according to the friction cost approach, and (4) analysis from the health care perspective.

## Results

The enrollment of the patients started October 2011. The follow-up period will be completed in August 2014. Data cleaning or analysis has not begun as of this article’s submission.

## Discussion

### Targeting Two Levels

This paper outlines the methodology of a stepped wedge cluster randomized trial to evaluate the cost effectiveness of a care program designed to improve postoperative recovery compared to the usual care. The intervention care program targets two levels: (1) the level of the hospital, and (2) the level of the patient. At the level of the hospital, the newly developed guidelines will be distributed among the clinical staff in order to stimulate evidence-based patient education at the time of discharge. At the patient level, access to an eHealth intervention is provided with tailored convalescence recommendations, and an occupational intervention is available, for those patients at risk of prolonged sick leave, for additional guidance.

### What This Study Will Add

The combination of increasing demands on the health care system and the limited health care budget designates a need to enhance the cost effectiveness of our health care system. The introduction of minimally invasive techniques in the last two decades has led to savings in in-hospital care due to shorter lengths of hospital stay, despite higher operative costs, longer operation time, and more expensive equipment [64-66]. However, early discharge does not necessarily lead to enhanced recovery, as postoperative recovery at home requires a different organization of perioperative care as well, such as preoperative patient education, including the deliverance of evidence-based standardized convalescence recommendations [6,8,9,12,67-70]. As far as we know, our care program is the first intervention developed, and being thoroughly evaluated, that anticipates this transition of perioperative care to the home setting. Second, the utilization of innovative eHealth technologies will limit the workload of involved health care professionals, anticipating a personnel shortage in the health care sector due to a shrinkage of the working population in the near future [71]. Finally, our trial will be one of few that conducted an economic evaluation from a societal perspective, not only taking into account solely direct medical costs—which are important for the hospital perspective—but also including costs associated with postoperative health care utilization and productivity losses due to absenteeism and presenteeism after discharge.

### Strengths and Limitations

The main strength of the present study is the choice for a stepped wedge cluster randomized trial. The contamination between study arms is prevented by the cluster design. In addition, the stepped wedge approach enables us to study the implementation process carefully, and gain valuable insight into the facilitators and barriers toward future implementation of the intervention program [72]. Because the crossover of the design is unidirectional, the intervention is not withdrawn from the hospitals during the trial. This is particularly convenient, as our previous trial supports our hypothesis that the care program will lead to enhanced postoperative recovery [73]. Finally, there is a statistical advantage to the stepped wedge approach because the intervention effect is estimated not only by between cluster comparisons, as in a parallel group design, but also by within cluster comparisons, limiting the risk of confounding and increasing statistical power [36,74].

This study also has limitations. First of all, randomized studies without blinding have higher risks of (selection) bias. A second limitation of this study might be the fact that some of the hospitals have already participated in the earlier trial in 2010. The existing knowledge about the convalescence recommendations could be a source of contamination for the current study, and could lead to an under estimation of the care program effect.

### Generalizability

The generalizability of this study will be high, due to the pragmatic study design. In order for procedures to be similar to clinical practice, interference of the research team will be minimized during the trial. The wide diversity of participating (7 teaching, 1 academic, and 1 nonteaching) hospitals, will also contribute to a heterogeneous sample of patients being enrolled in this study, enhancing generalizability. However, we should also be aware of factors that could possibly limit the external validity. A typical feature of eHealth interventions is the risk of selection bias toward the higher educated participants as compared to the general population. Moreover, as the care program was developed in the Dutch setting, and especially tailored to Dutch patients, generalizability of the results of this trial to other countries will be unknown, due to differences in social and health care systems.

### Policy Implications

The results of this cost effectiveness study will enable health care policy makers to decide about future implementation of the care program on a broad scale in the Netherlands. In the case that the care program under study is proven to be cost effective, this will have considerable impact. Most importantly, the financial burden on society due to prolonged sick leave following benign gynecological surgery will be substantially reduced. Also, the individual patients will benefit through increased quality of life, and employers will profit because of a decline in absenteeism rates. Moreover, for health care professionals, the care program will be an asset, as it will lead to better organized and more efficient care. Finally, the care program has the potential to maximize the beneficial effects of other recovery enhancing strategies, such as the use of minimally invasive surgery.

## Multimedia Appendix 1

Screenshots of ikherstel.

## Multimedia Appendix 2

CONSORT-EHEALTH checklist V1.6.2 [34].

## Multimedia Appendix 3

Grant Proposal.

## Multimedia Appendix 4

Letter of Grant Approval.

## Abbreviations

CONSORT: CONsolidated Standards Of Reporting Trials
DMQ: Dutch Musculoskeletal Questionnaire
EQ-5D: EuroQol-5D
GP: general physician
GSES: General Self-Efficacy Scale
ICER: incremental cost effectiveness ratio
IQR: interquartile range
JCQ: Job Content Questionnaire
LAS: laparoscopic adnexal surgery
OP: occupational physician
OT: occupational therapist
PMS: Pearlin Mastery Scale
RI-10: Recovery Index-10
RTW: return to work
SF-36: Short-Form Health Survey
TAH: total abdominal hysterectomy
TLH: total laparoscopic hysterectomy
TP: time period
VAS: Visual Analogue Scale
VH: vaginal hysterectomy
