# Supplementary material for: The Cost Effectiveness of a Tailored, Web-Based Care Program to Enhance Postoperative Recovery in Gynecologic Patients in Comparison With Usual Care: Protocol of a Stepped Wedge Cluster Randomized Controlled Trial
Source: JMIR Res Protoc. 2014 Jun 18;3(2):e30. doi: 10.2196/resprot.3236 (PMC4090379; doi:10.2196/resprot.3236)
Supplement: Supplementary file 1 [file resprot_v3i2e30_app1.ppt]

## Slide 1
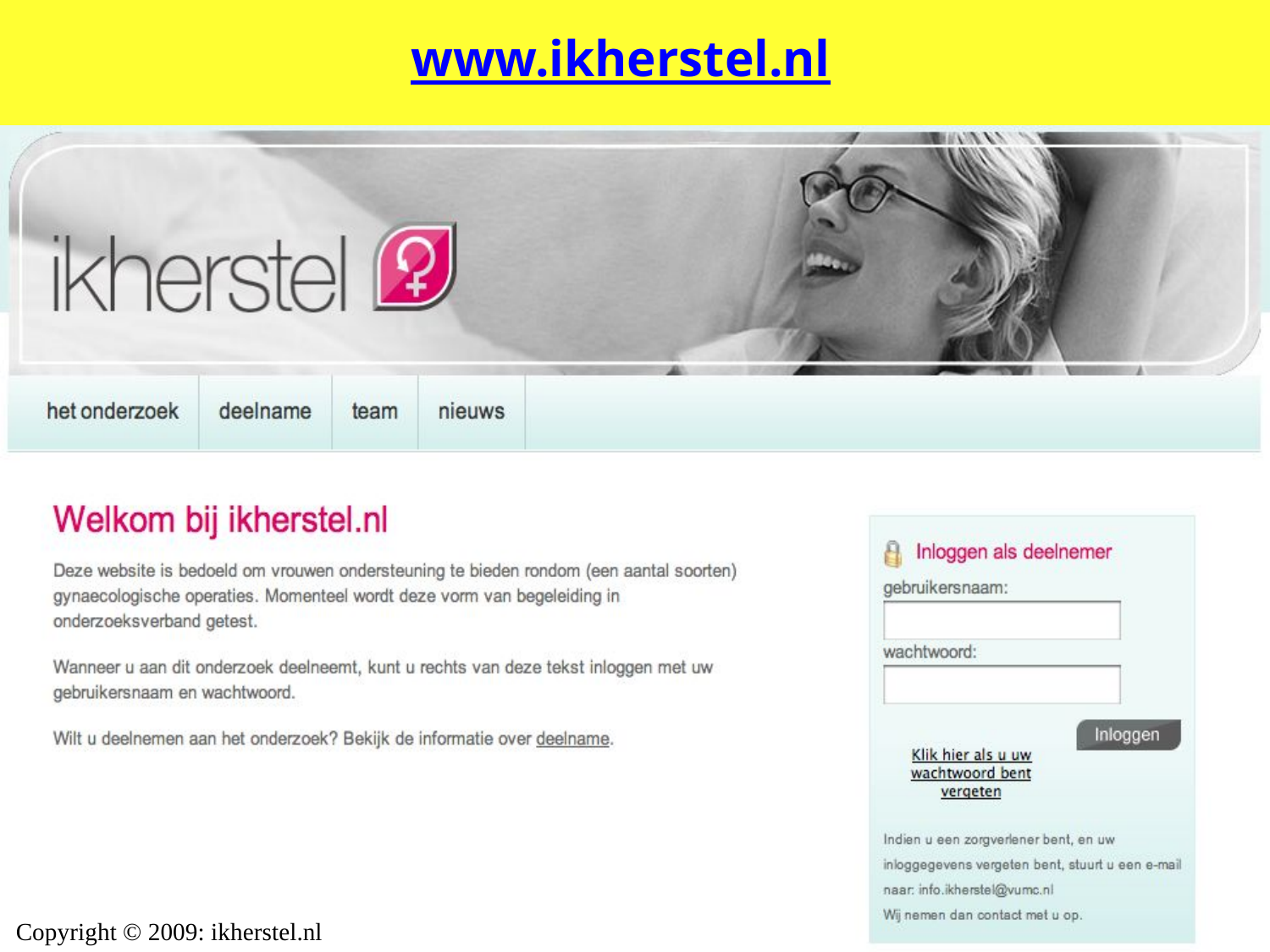

# www.ikherstel.nl
Copyright © 2009: ikherstel.nl

## Slide 2
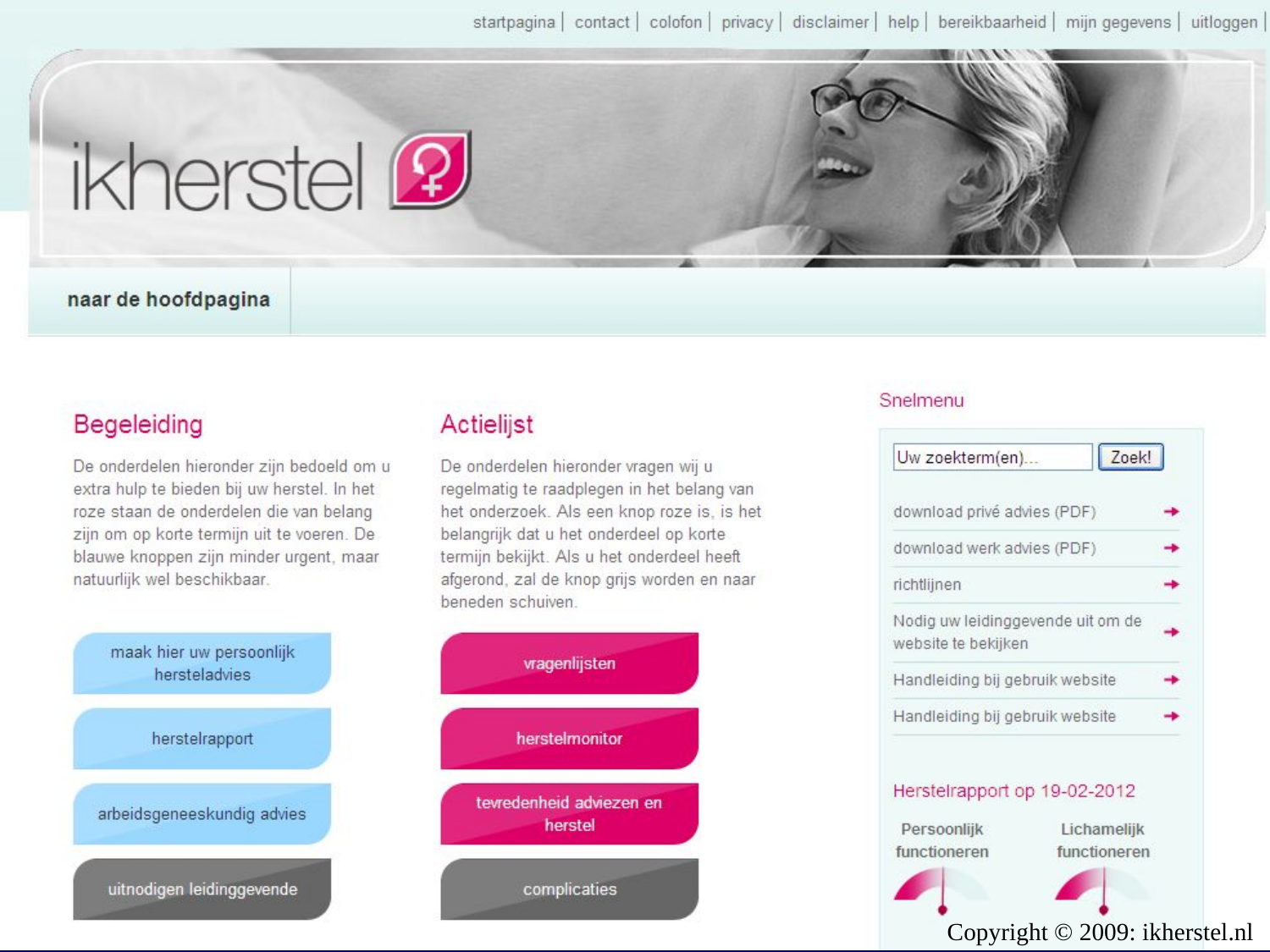

Copyright © 2009: ikherstel.nl

## Slide 3
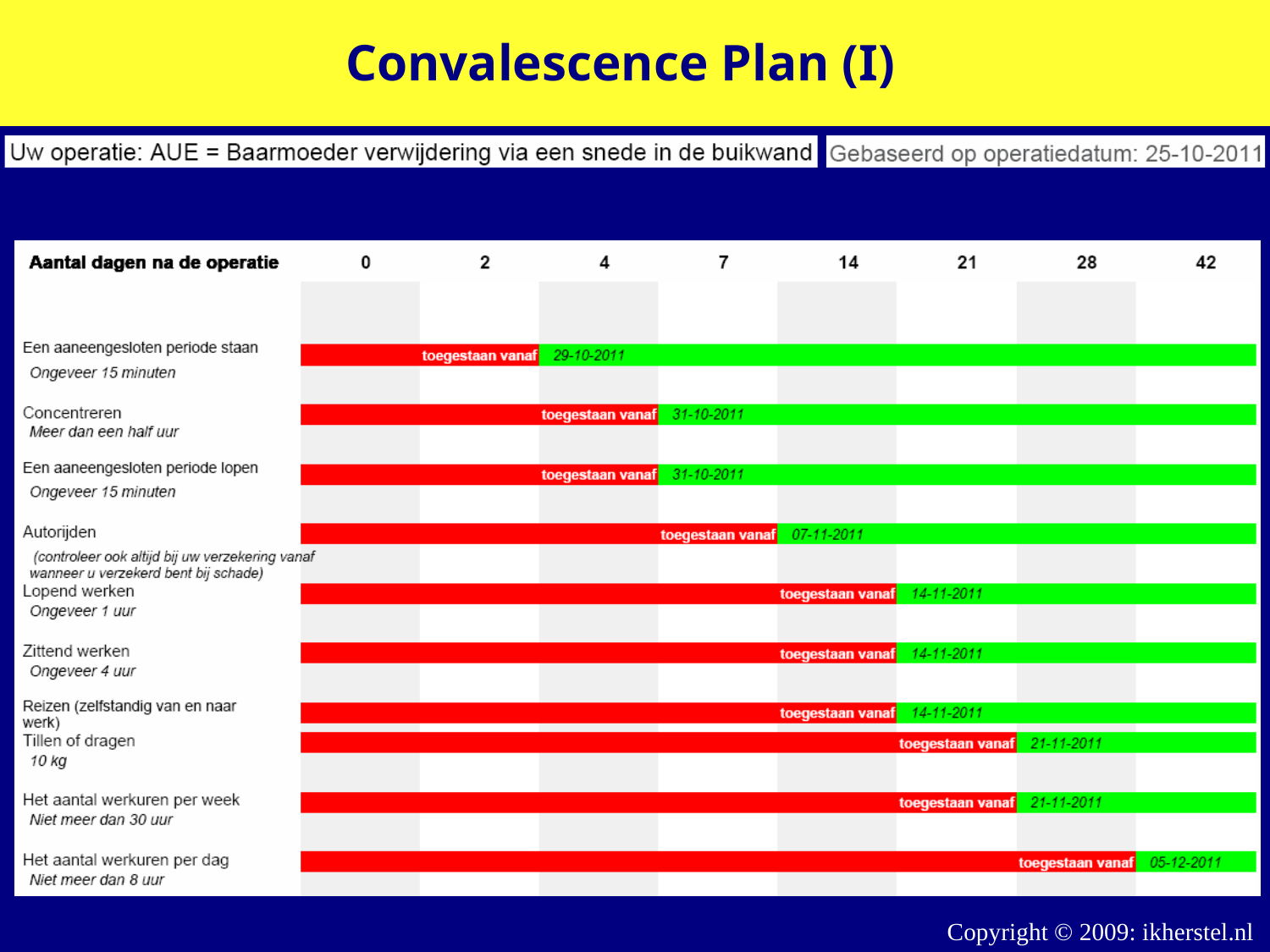

# Convalescence Plan (I)
Copyright © 2009: ikherstel.nl

## Slide 4
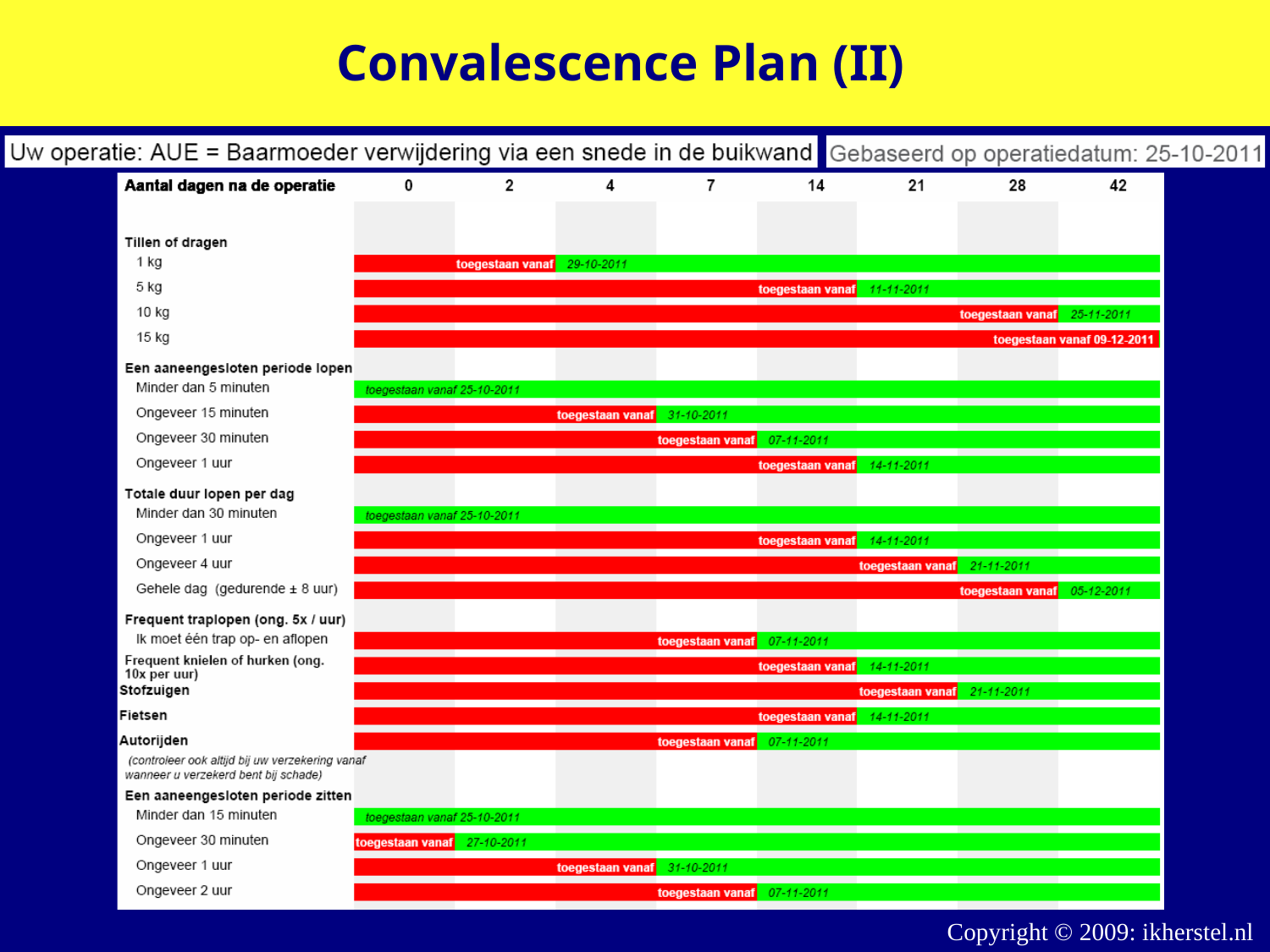

# Convalescence Plan (II)
Copyright © 2009: ikherstel.nl

## Slide 5
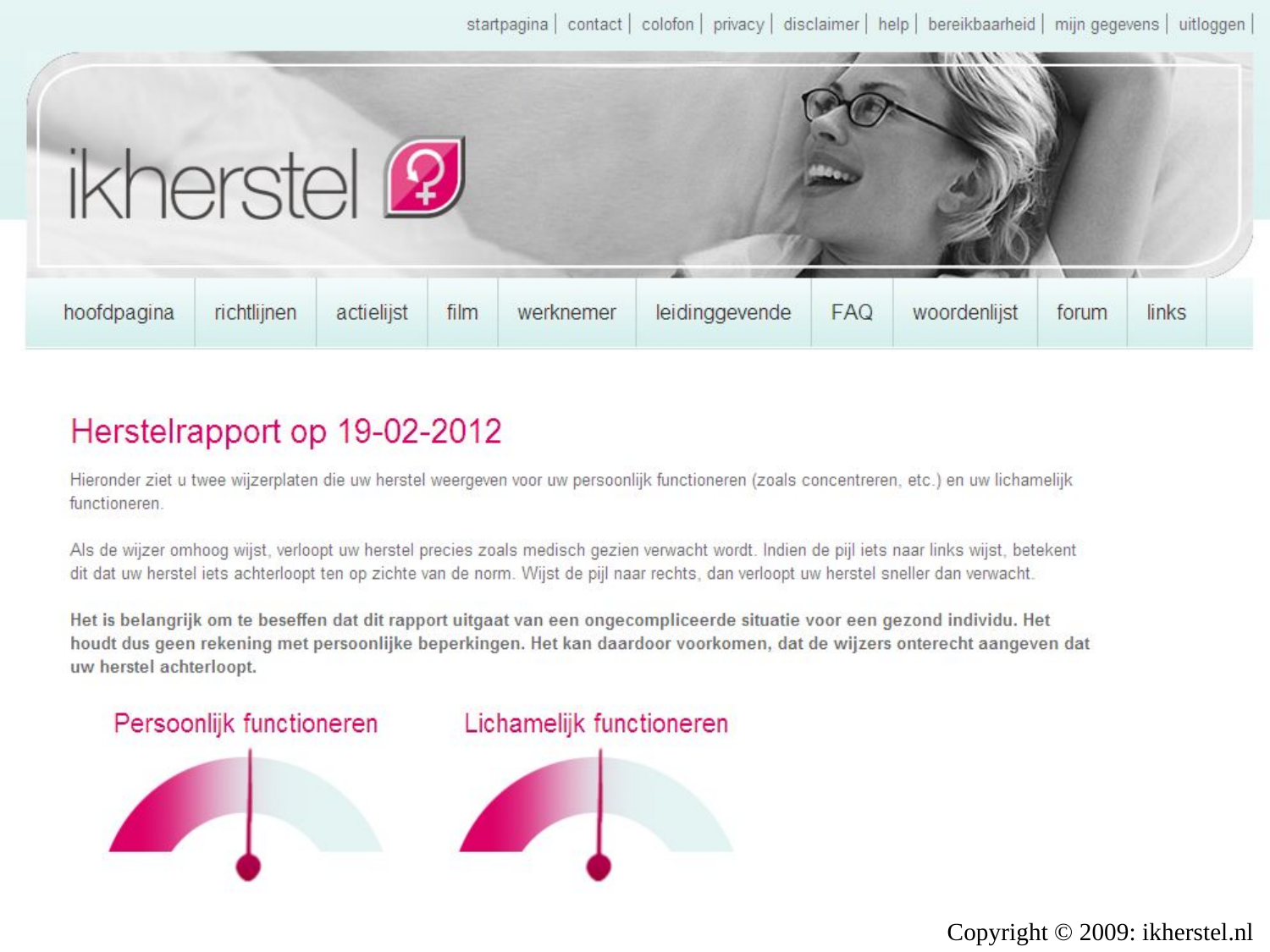

Copyright © 2009: ikherstel.nl

## Slide 6
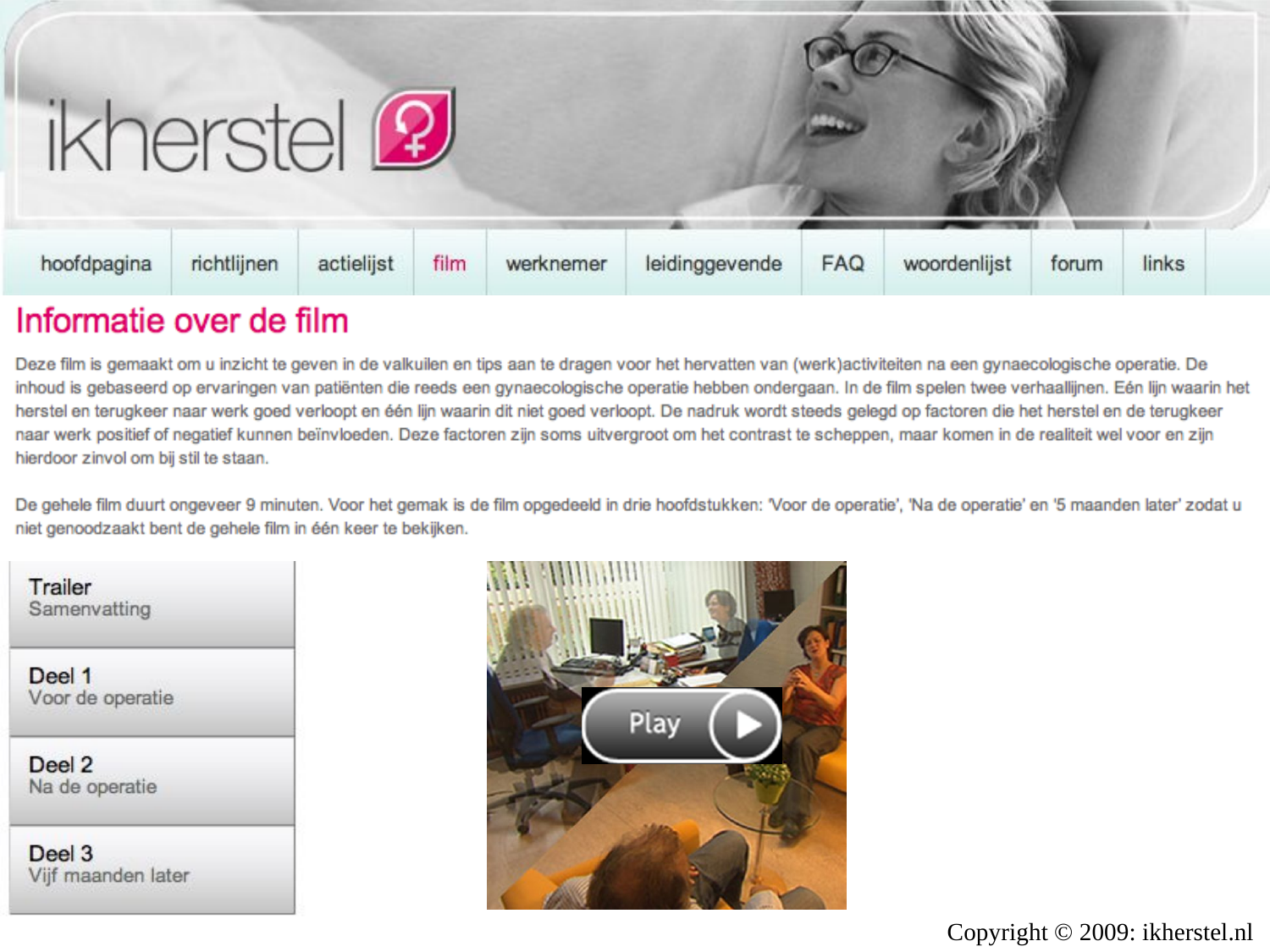

Copyright © 2009: ikherstel.nl
