# Supplementary material for: The Cost Effectiveness of a Tailored, Web-Based Care Program to Enhance Postoperative Recovery in Gynecologic Patients in Comparison With Usual Care: Protocol of a Stepped Wedge Cluster Randomized Controlled Trial
Source: JMIR Res Protoc. 2014 Jun 18;3(2):e30. doi: 10.2196/resprot.3236 (PMC4090379; doi:10.2196/resprot.3236)
Supplement: Supplementary file 2 [file resprot_v3i2e30_app2.pdf]

CONSORT-EHEALTH Checklist V1.6.2 Report

(based on CONSORT-EHEALTH V1.6), available at [http://tinyurl.com/consort-ehealth-v1-6].

3236

Date completed

3/3/2014 8:53:46

by

Esther Bouwsma

Cost-effectiveness of a tailored, web-based care program to enhance postoperative recovery in gynaecologic patients in comparison with usual care; research protocol of a stepped wedge cluster randomized trial

TITLE

1a-i) Identify the mode of delivery in the title

title includes: “web-based care program”

1a-ii) Non-web-based components or important co-interventions in title

No. Non-web-based component of the care program (the occupational intervention) is not delivered to all patients in intervention group (only for patients at risk of prolonged sick leave).

1a-iii) Primary condition or target group in the title

title includes: “gynecologic patients”

ABSTRACT

1b-i) Key features/functionalities/components of the intervention and comparator in the METHODS section of the ABSTRACT

Key features of intervention: “The care program under study, designed to improve convalescence and to prevent delayed return to work, targets two levels. At hospital level, guidelines will be distributed among clinical staff in order to stimulate evidence-based patient education. At patient level, additional perioperative guidance is provided by means of an eHealth intervention equipping patients with tailored convalescence advice and an occupational intervention is available for those patients at risk of prolonged sick leave."

Comparator: “…compared to usual care.”

1b-ii) Level of human involvement in the METHODS section of the ABSTRACT

No. However, it is in the main body of text.
"Access to the webportal will be given to the patients approximately 2 to 4 weeks prior to surgery by the research team, by providing an username and temporary password."

1b-iii) Open vs. closed, web-based (self-assessment) vs. face-to-face assessments in the METHODS section of the ABSTRACT

Recruitment: “… nine participating teaching hospitals, from which the patients are recruited.”
Assessment: “Data will be collected by means of self-reported electronic questionnaires at baseline and 2 weeks, 6 weeks, 12 weeks, 26 weeks and 52 weeks follow-up.”

1b-iv) RESULTS section in abstract must contain use data

n/a (research protocol)

1b-v) CONCLUSIONS/DISCUSSION in abstract for negative trials

n/a (research protocol)

INTRODUCTION

2a-i) Problem and the type of system/solution

Problem: “the financial burden on society due to delayed convalescence after gynecological surgery is substantial.”
Solution: “In order to reduce unnecessary delayed recovery and concurrently decrease costs associated with prolonged sick leave following gynecological surgery, our research group started working on an innovative strategy to optimize perioperative care in 2008.”
Goals: “The care program provides guidance to patients from the moment the surgery is planned until full resumption of all activities – including return to work – and encourages patients to take an active role in their own recovery.”

2a-ii) Scientific background, rationale: What is known about the (type of) system

The care program was subject to an effect-evaluation as well as a process-evaluation in 2010 [26]. While the effectiveness study among 215 patients showed a positive effect on the outcomes return to work, quality of life and perceived pain [27], the process evaluation showed some room for improvement [28].

METHODS

3a) CONSORT: Description of trial design (such as parallel, factorial) including allocation ratio

“The primary objective of this current study is to conduct an economic evaluation of the care program compared to usual care.”
“We hypothesize that the intervention program will reduce the financial burden of sick leave after gynecological surgery by decreasing absenteeism from paid work.”

3b) CONSORT: Important changes to methods after trial commencement (such as eligibility criteria), with reasons

n/a (research protocol)

3b-i) Bug fixes, Downtimes, Content Changes

n/a (research protocol)

4a) CONSORT: Eligibility criteria for participants

“Table 1 lists an overview of all eligibility criteria.”

4a-i) Computer / Internet literacy

“(…) factors interfering with the e-health intervention (computer or internet illiteracy) (…) serve as exclusion criteria.”

4a-ii) Open vs. closed, web-based vs. face-to-face assessments:

“Recruitment of patients will find place in all participating hospitals.”
“It consists of two steps: 1) access to an interactive eHealth intervention for all patients and 2) an additional occupational intervention for those patients at risk for prolonged sick leave.” The latter find place by telephone-contact.

4a-iii) Information giving during recruitment

“When patients are scheduled for laparoscopic surgery and / or hysterectomy, they will receive a letter about the study on behalf of their gynaecologist. The letter includes detailed information about the trial. In addition it is explained that someone from the research-team will make contact by telephone after one week to evaluate willingness to participate and answer questions if necessary. If the patient does not wish to be contacted, she can return an included reply card, or send an email to a specified email address. When contact is made and the patient is willing to participate, eligibility is assessed. Eligible patients are then requested to return a signed informed consent, which is also attached to the information letter.”

4b) CONSORT: Settings and locations where the data were collected

"The clusters in this trial consist of nine hospitals in the surroundings of Amsterdam, the capital of the Netherlands. Hospitals were eligible if they performed at least 100 hysterectomies or laparoscopic adnexal surgeries yearly and were located within 50 km of the VU University Medical Center. Clusters were enrolled by the research-team before the start of the trial. In an attempt to select a heterogeneous sample of hospitals, we included 1 university hospital, 7 teaching hospitals and 1 non-teaching hospital."

4b-i) Report if outcomes were (self-)assessed through online questionnaires

"Data will be collected by means of self-reported electronic questionnaires [58] before surgery and 2 weeks (T1), 6 weeks (T2), 12 weeks (T3), 26 weeks (T4) and 52 weeks (T5) after surgery."

4b-ii) Report how institutional affiliations are displayed

n/a. Patients are recruited from participating hospitals.

5) CONSORT: Describe the interventions for each group with sufficient details to allow replication, including how and when they were actually administered

5-i) Mention names, credential, affiliations of the developers, sponsors, and owners

"EVB, AV, HAB, JRA and JAH are the developers of www.ikherstel.nl."

"This study is funded by ZonMw, an organization for health research and development in the Netherlands (project numbers 150020037, 171102015 and 92003590)."

The web-based intervention was realized by D. Stomp, LF8 – elevate internet (http://www.lf8.nl).

5-ii) Describe the history/development process

"Simultaneously, a multidisciplinary care program was developed [21;22] consisting of an interactive e-health intervention and – for those patients at risk of prolonged sick leave – an occupational intervention."

5-iii) Revisions and updating

Table 2 summarizes the most important tools of the e-health intervention."

"Italic text present tools that were modified since the last evaluation of the webportal"

5-iv) Quality assurance methods

n/a

5-v) Ensure replicability by publishing the source code, and/or providing screenshots/screen-capture video, and/or providing flowcharts of the algorithms used

"Screenshots of the webportal are included as a Multimedia Appendix (see Multimedia Appendix 1)."

5-vi) Digital preservation

"http://www.ikherstel.nl"

Demo-logins are available for those interested (contact: ev.bouwsma@vumc.nl).

5-vii) Access

"Access to the webportal will be given to the patients approximately 2 to 4 weeks prior to surgery by the research team, by providing an username and temporary password."

5-viii) Mode of delivery, features/functionalities/components of the intervention and comparator, and the theoretical framework

"Table 2 summarizes the most important tools of the e-health intervention."

"The Attitude, Social influence and Self-Efficacy model was used as a theoretical framework for determinants of behavior regarding return to work [36,37]."

5-ix) Describe use parameters

"Instructions are given by email (...) "

5-x) Clarify the level of human involvement

"Access to the webportal will be given to the patients approximately 2 to 4 weeks prior to surgery by the research team, by providing an username and temporary password. Instructions are given by email and it is explained that if patients require assistance, they can contact the research team by phone or e-mail."

5-xi) Report any prompts/reminders used

"If patients fail to login, an automatic reminder is sent to them one week before surgery, to remind them about the webportal and its functionalities." Moreover, patients receive an automatic reminder (email) to fill out the recovery monitor at 2, 4, 7, 14, 28, 56 and 84 days after surgery.

5-xii) Describe any co-interventions (incl. training/support)

"Co-interventions during the intervention-period cannot always be avoided. However, we will be able to determine whether patients received co-interventions by means of the monthly cost-diaries."

6a) CONSORT: Completely defined pre-specified primary and secondary outcome measures, including how and when they were assessed

"Table 3 provides an overview of all outcome measures and assessment instruments used in this trial."

6a-i) Online questionnaires: describe if they were validated for online use and apply CHERRIES items to describe how the questionnaires were designed/deployed

"Not all instruments have been validated for online use."

6a-ii) Describe whether and how "use" (including intensity of use/dosage) was defined/measured/monitored

"User authentication will make it possible to analyze website activity for each individual participant (visit duration, number of sessions, number and details of pages visited).."

"In the instruction email, patients are recommended to generate such a plan at least once, preferably before surgery."

6a-iii) Describe whether, how, and when qualitative feedback from participants was obtained

During the development of the intervention the engagement of patient was prompted through focus groups [25]. Focus groups with participants will be held in 2014 to assess facilitators and barriers to future implementation.

6b) CONSORT: Any changes to trial outcomes after the trial commenced, with reasons

n/a (research protocol)

7a) CONSORT: How sample size was determined

7a-i) Describe whether and how expected attrition was taken into account when calculating the sample size

"(...) taking into account a drop-out rate of 10% (...)"

7b) CONSORT: When applicable, explanation of any interim analyses and stopping guidelines

n/a

8a) CONSORT: Method used to generate the random allocation sequence

"Randomization takes place at the level of the clusters and determines the order in which the intervention-program is implemented in the participating hospitals. Randomization will be performed by a statistician using a computer generated list of random numbers."

8b) CONSORT: Type of randomisation; details of any restriction (such as blocking and block size)

n/a

9) CONSORT: Mechanism used to implement the random allocation sequence (such as sequentially numbered containers), describing any steps taken to conceal the sequence until interventions were assigned

"Patients are informed about the allocation of treatment by the research team after informed consent and completion of the first questionnaire before surgery. As treatment allocation depends on the scheduled date of surgery and the implementation-phase of the hospital in which they are being operated, it is predetermined for each participant, potentially causing selection bias. To minimize the risk of selection bias, participants will not be informed about the study design and will be counseled as if they have equal chances between receiving usual care or the intervention-program. For this reason, counseling will be done by the research team, rather than by their own physician, who might be e.g. more willing to include patients during the intervention-phase than during the control-phase. Moreover, physicians will be blinded to the randomization schedule and will only be informed about the start of the intervention-phase approximately one month before the actual implementation. Once the intervention-phase has started, the importance of not communicating this information with potential patients will be emphasized."

10) CONSORT: Who generated the random allocation sequence, who enrolled participants, and who assigned participants to interventions

see subitem 9

11a) CONSORT: Blinding - If done, who was blinded after assignment to interventions (for example, participants, care providers, those assessing outcomes) and how

11a-i) Specify who was blinded, and who wasn't

"Participants, care providers and researchers cannot be blinded for the allocated treatment. However, analysis of the data by the researcher will be blind as all patients receive their own study-code, under which their data is stored in the database. Information bias due to assessors being aware of the allocation will be prevented because the assessment of outcomes is measured through self-reported questionnaires."

11a-ii) Discuss e.g., whether participants knew which intervention was the "intervention of interest" and which one was the "comparator"

n/a. Patients will not be blinded

11b) CONSORT: If relevant, description of the similarity of interventions

n/a

12a) CONSORT: Statistical methods used to compare groups for primary and secondary outcomes

"For the primary outcome, the duration of sick leave until full sustainable RTW, Cox regression analyses will be used to investigate the intervention effect. Both crude and adjusted analyses will be performed. In the adjusted analyses the following variables will be used as covariates: (1) hospital, to adjust for clustering (random gamma effect), (2) type of surgery performed, (3) time period, to adjust for naturally occurring changes over time irrespective of the intervention, (4) optionally, (time period) x (intervention) interaction term, to adjust for time effects (the longer the care program is implemented, the more effective it might be). "

"Differences in secondary outcomes will be assessed using generalized linear longitudinal mixed models. All available measurements (2weeks, 6weeks, 12 weeks, 26 weeks and 52 weeks) will be used and baseline scores will be used as covariates, as well as the hospital and the type of surgery (random effect)."

12a-i) Imputation techniques to deal with attrition / missing values

"Missing cost and effect data will be imputed using multiple imputation [59]. The imputation will include variables that are related to missing data or the outcome measure, and variables that differ at baseline between the groups."

12b) CONSORT: Methods for additional analyses, such as subgroup analyses and adjusted analyses

"To assess whether protocol deviations caused bias, a per-protocol analysis will be performed and the results will be compared to the intention-to-treat analyses. In addition, several subgroup analyses will be performed. Predefined subgroups will be: (1) hysterectomy (TAH, VH, TLH), (2) minimal invasive hysterectomy (VH, TLH), (3) abdominal hysterectomy only, and (4) laparoscopic adnexal surgery only. "To assess the robustness of results, several secondary economic analyses will be performed: (1) complete case analysis, (2) per-protocol analysis, (3) analysis with costs calculated according to the friction cost approach, and (4) analysis from the healthcare perspective."

RESULTS

13a) CONSORT: For each group, the numbers of participants who were randomly assigned, received intended treatment, and were analysed for the primary outcome

n/a (research protocol)

13b) CONSORT: For each group, losses and exclusions after randomisation, together with reasons

n/a (research protocol)

13b-i) Attrition diagram

n/a (research protocol)

14a) CONSORT: Dates defining the periods of recruitment and follow-up

n/a (research protocol)

14a-i) Indicate if critical "secular events" fell into the study period

n/a (research protocol)

14b) CONSORT: Why the trial ended or was stopped (early)

n/a (research protocol)

15) CONSORT: A table showing baseline demographic and clinical characteristics for each group

n/a (research protocol)

15-i) Report demographics associated with digital divide issues

n/a (research protocol)

16a) CONSORT: For each group, number of participants (denominator) included in each analysis and whether the analysis was by original assigned groups

16-i) Report multiple "denominators" and provide definitions

n/a (research protocol)

16-ii) Primary analysis should be intent-to-treat

n/a (research protocol)

17a) CONSORT: For each primary and secondary outcome, results for each group, and the estimated effect size and its precision (such as 95% confidence interval)

n/a (research protocol)

17a-i) Presentation of process outcomes such as metrics of use and intensity of use

n/a (research protocol)

17b) CONSORT: For binary outcomes, presentation of both absolute and relative effect sizes is recommended

n/a (research protocol)

18) CONSORT: Results of any other analyses performed, including subgroup analyses and adjusted analyses, distinguishing pre-specified from exploratory

n/a (research protocol)

18-i) Subgroup analysis of comparing only users
n/a (research protocol)
19) CONSORT: All important harms or unintended effects in each group
n/a (research protocol)
19-i) Include privacy breaches, technical problems
n/a (research protocol)
19-ii) Include qualitative feedback from participants or observations from staff/researchers
n/a (research protocol)

DISCUSSION

20) CONSORT: Trial limitations, addressing sources of potential bias, imprecision, multiplicity of analyses
20-i) Typical limitations in ehealth trials
"A typical feature of eHealth interventions is the risk of selection bias towards higher educated participants compared to the general population."
21) CONSORT: Generalisability (external validity, applicability) of the trial findings
21-i) Generalizability to other populations
"Generalisability of this study will be high, due to the pragmatic study design. In order for procedures to be similar to clinical practice, interference of the research team will be minimized during the trial. The wide diversity of participating (7 teaching, 1 academic and 1 non-teaching) hospitals, will also contribute to a heterogeneous sample of patients being enrolled in this study, enhancing generalisability. However, we should also be aware of factors that possibly limit external validity. A typical feature of eHealth interventions is the risk of selection bias towards higher educated participants compared to the general population. Moreover, as the care program was developed in the Dutch setting and especially tailored to Dutch patients, generalisability of the results of this trial to other countries will be unknown, due to differences in social- and health care systems. "
21-ii) Discuss if there were elements in the RCT that would be different in a routine application setting
"In order for procedures to be similar to clinical practice, interference of the research team will be minimized during the trial."
22) CONSORT: Interpretation consistent with results, balancing benefits and harms, and considering other relevant evidence
22-i) Restate study questions and summarize the answers suggested by the data, starting with primary outcomes and process outcomes (use)
n/a (research protocol)
22-ii) Highlight unanswered new questions, suggest future research
n/a (research protocol)

Other information

23) CONSORT: Registration number and name of trial registry
Nederlands Trial Register: NTR2933
24) CONSORT: Where the full trial protocol can be accessed, if available
Grant-proposal updated as supplementary files during submission-process.
25) CONSORT: Sources of funding and other support (such as supply of drugs), role of funders
"This study is funded by ZonMw, an organization for health research and development in the Netherlands (project numbers 150020037, 171102015 and 92003590)."
X26-i) Comment on ethics committee approval
"This study protocol was approved by the Institutional Review Boards of all participating hospitals. Informed consent was obtained from all patients."
x26-ii) Outline informed consent procedures
"When contact is made and the patient is willing to participate, eligibility is assessed. Eligible patients are then requested to return a signed informed consent, which is also attached to the information letter."
X26-iii) Safety and security procedures
"… all patients receive their own study-code, under which their data is stored in the database."
X27-i) State the relation of the study team towards the system being evaluated
"EVB, AV, HAB, JRA and JAH are the developers of www.ikherstel.nl."
