# Supplementary material for: The Cost Effectiveness of a Tailored, Web-Based Care Program to Enhance Postoperative Recovery in Gynecologic Patients in Comparison With Usual Care: Protocol of a Stepped Wedge Cluster Randomized Controlled Trial
Source: JMIR Res Protoc. 2014 Jun 18;3(2):e30. doi: 10.2196/resprot.3236 (PMC4090379; doi:10.2196/resprot.3236)
Supplement: Supplementary file 3 [file resprot_v3i2e30_app3.pdf]

Algemene gegevens / General Information

Programma / Programme	:	
Subsidieronde / Subsidy round	:	**Ronde 11 Grant application E&K**
Projecttitel / Project title	:	**The cost-effectiveness of a transmural peri-operative care program for gynaecology**
Projecttaal / Project language	:	**Engels / English**
Geplande startdatum / Planned start date	:	**01-01-2011**
Geplande duur / Planned duration	:	**48 maanden / months**
Datum indienen / Date of application	:	**17-08-2010**
Projecttype / Project type	:	**Toegepast onderzoek / Applied research**
Vervolg eerder ZonMw-project / Continuation previously funded project ZonMw	:	**Ja / Yes**
Eerder gehonoreerd projecttype / Project type of previously funded project	:	**Ontwikkelproject / Develop project**

Aanvrager / Applicant

Dr. J.A.F Huirne
T: +31 20 4444851 | *F:* | *E:* j.huirne@vumc.nl

VU medisch centrum
Verloskunde en Gynaecologie
Endoscopische Gynaecologie
De Boelelaan 1117
1081 HV AMSTERDAM

Projectleden / Project members

Prof. dr. J.R. Anema (Mede aanvrager)
Functie / Position: bedrijfsarts, verzekeringsarts, senior-onderzoeker | *Opleiding / Education:*
Studierichting / Subject:
T: +31 20 4446495 | *F:* +31 20 4448387 | *E:* h.anema@vumc.nl

VU Medisch Centrum
EMGO Institute for Health and Care Research
Public and Occupational Health
Postbus 7057
1007 MB AMSTERDAM

Prof. dr. H.A.M. Brölmann (Mede projectleider)
Functie / Position: Hoofd afdeling Gynaecologie | *Opleiding / Education:*
Studierichting / Subject:
T: +31 20 4444851 | *F:* +31 20 4448387 | *E:* h.brolmann@vumc.nl

VU medisch centrum
Verloskunde en Gynaecologie
Endoscopische Gynaecologie
De Boelelaan 1117
1081 HV AMSTERDAM

Dr. J.A.F Huirne (Projectleider en penvoerder)
Functie / Position: Gynaecoloog, senior onderzoeker | *Opleiding / Education:*
Studierichting / Subject:
T: +31 20 4444851 | *F:* +31 20-4443333 | *E:* j.huirne@vumc.nl

VU medisch centrum
Verloskunde en Gynaecologie

Endoscopische Gynaecologie
De Boelelaan 1117
1081 HV AMSTERDAM

Prof. dr. B.W. Mol (Projectadviseur)
Functie / Position: gynaecoloog, voorzitter commissie richtlijnen NVOG | *Opleiding / Education:*
Studierichting / Subject:
T: 030- 2823 812 | *F:* | *E:* info@nvog.nl

Nederlandse Vereniging voor Obstetrie en Gynaecologie
Commissie richtlijnen
GBS richtlijn
Postbus 20061
3502 LB UTRECHT

Prof. dr. T.J.F. Savelkoul (Bestuurlijk verantwoordelijke)
Functie / Position: Vice-voorzitter raad van bestuur VUmc | *Opleiding / Education:*
Studierichting / Subject:
T: 020-4444444 | *F:* | *E:* tjf.savelkoul@vumc.nl

VU medisch centrum
Raad van Bestuur
Postbus 7057
1007 MB AMSTERDAM

Prof. dr. M.W. van Tulder (Projectadviseur)
Functie / Position: Professor in Health Technology Assessment | *Opleiding / Education:*
Studierichting / Subject:
T: 020-4448178 | *F:* | *E:* mw.vantulder@vumc.nl

VU medisch centrum
EMGO Institute for Health and Care Research
Postbus 7057
1007 MB AMSTERDAM

Prof. dr. W. van Mechelen (Begeleider)
Functie / Position: Bedrijfsarts, hoofd Public and Occupational health | *Opleiding / Education:*
Studierichting / Subject:
T: +31 20 4448206 | *F:* +31 20 4448387 | *E:* w.vanmechelen@vumc.nl

VU Medisch Centrum
EMGO Institute for Health and Care Research
Public and Occupational Health
Postbus 7057
1007 MB AMSTERDAM

Projectgegevens / Project information

Aandachtsgebieden / Focus

Optimalisatie van organisatie van zorg
- Kennisoverdracht
- Optimalisatie afstemming en samenwerking

Optimalisatie van therapie
- Follow-up
- Minimaal invasieve behandeling
- Multidisciplinaire behandeling

Samenvatting / Summary

HEALTHPROBLEM
Peri-operative gynaecological care is fragmented, poorly coordinated and often conflicting advices are provided by concerned care providers (gynaecologists, general practitioners(PGs), occupations physicians (OPs), insurance physicians IPs). Resumption of (work)activities takes much longer than expected after gynaecological surgery, irrespective the level of invasiveness [21,25]. Long sickleave has proven to result in increased risk of work disability and general and mental health problems. Patients-(NPCF,BBV&W) and professional organizations (KNMG, NVOG, NHG, NVVG, NVAB, Dutch Health Council) identified these problems after all kind of surgical procedures and underline the need for optimization of current peri-operative care (see attachments).

OBJECTIVE
To evaluate the cost-effectiveness and cost-utility of an innovative peri-operative transmural care program conducted in

patients undergoing gynaecological surgery.

TRANSMURAL PERI-OPERATIVE CARE PROGRAM
To improve peri-operative gynaecological care, we developed in a previous project (ZonMW,KKCZ) a multidisciplinary care program, containing the use of an interactive weblog and a workplace intervention (participative ergonomy).
The interactive weblog provides patient-tailored detailed instructions on the resumption of (work)activities. These recommendations are based on consensus achieved among gynaecologists, GPs, OPs and GPs using a structural consensus method, including a systematic review. The weblog additionally provides tools to improved self-empowerment and to improve the communication between patients, care-providers and employers, preventing conflicting recommendations. Patients' recovery can be closely monitored by the weblog, allowing the application of a very successful workplace (participative ergonomy) intervention to improve patients' recovery and reduce sick-leave.

POTENTIAL BENEFITS ON PATIENTS RECOVERY
Improved recovery and accelerated resumption of(work)activities. Resumption of work contributes significantly to QOL and will prevent disability due to general and mental health problems and associated financial deprivation.

POTENTIAL REDUCTION OF DIRECT AND INDIRECT COSTS
a) Reduced medical consultation due to improved care-coordination and communication.
b) Reduced costs due to less days with activity loss: yearly > 20.000 women receive a hysterectomy or laparoscopic adnexal surgery in the Netherlands. Based on studies, we estimate that a reduction of 400.000 lost days/year(4wks x20.000 operations) will be feasible for these procedures in the Netherlands.
c) Sickleave and associated health problems are strongly associated with increased medical consumption and compensation costs.
d) Comparable recommendations may be applicable for all surgical procedures in urology-, surgery-, gynaecology-, orthopaedic-, plastic and cosmetic surgery, responsible for 1.087.139 procedures a year in the Netherlands.[41]

K&E
As a result of our previous project(ZonMw KKCZ project) recommendations for peri-operative provided care, including instructions for postoperative resumption of activities, have been formulated. After completion of the light version guideline it will be offered to all organizations representing specialists who apply peri-operative care and stimulate work-reintegration after gynaecological surgery (NVOG,NHG,NVAB,NVVG). These organizations will adjust these guidelines to their individual regulations after which it may be incorporated in official guidelines of these organizations. However the cost-effectiveness of the transmural peri-operative care program in comparison to usual care has yet to be established. In our opinion this should be performed on a short term before implementation of this innovative care program in daily practice. Early implementation of the innovative program makes proper comparison with "usual proviced care" difficult due to contamination.

STUDY POPULATION AND DESIGN
Patients: 280 women, scheduled for a hysterectomy or laparoscopic adnexal surgery will receive either "usual care" or the transmural peri-operative care program. Step-wedged randomized controlled trial, with sequential roll-out of the intervention in 7 centers. All start with usual care and every 2 months one centre will implement the intervention.

OUTCOME MEASURES
Primary outcome measures: 1)validated Recovery specific-QOL(RI-10)and 2)Return to work (RTW). Secondary outcome measures are generic QOL (SF36, euroQOL), satisfaction with provide care-program, direct and indirect costs 1x/month. Possible confounders (JCS) will be measured and process evaluation will be conducted.

ECONOMIC EVALUATION
Cost-effectiveness/utility analysis from the societal perspective, using bootstrapping.

PLANNING (months)
0-6: study organization phase
7-23: inclusion phase
8-35: follow-up phase
28-37: analytic phase
38-48: publications(5 papers, PhD-thesis)

Trefwoorden / Keywords

multidisciplinary; peri-operative; care-program;detailed instructions; ICT self-management; workplace intervention; recovery; re-integration

Samenwerking / Collaboration

Samenwerking tussen onderzoek en praktijk / Cooperation between research and practice:

Ja / Yes

Organisaties

Nederlands Huisartsen Genootschap

Postbus 3231
3502 GE UTRECHT

Nederlandse Vereniging voor Arbeids- en Bedrijfsgeneeskunde
Postbus 2113
3500 GC UTRECHT

Nederlandse Vereniging voor Obstetrie en Gynaecologie
Postbus 20075
3502 LB UTRECHT

Nederlandse Vereniging voor Verzekeringsgeneeskunde
Postbus 24095
3502 MB UTRECHT

Vereniging Nederlandse Patienten Consumenten Federatie
Patienten Praktijk
Postbus 3351
3502 GJ UTRECHT

Inhoud / Content

Probleemstelling / Problem definition

HEALTH PROBLEM
Resumption of work activities after gynaecological surgeries takes much longer than expected, irrespective surgical technique and level of invasiveness.[17,21,25,26]

Median sickleave in a prospective observational study in 60 women, after gynaecological procedures for benign conditions in exceeded 8 weeks[21,10]. Prolonged sickleave was only to a small extend effected by the level of invasiveness. Preliminary data of a larger observational study in 150 women performed by our study group, of which 130 completed the follow-up, confirm these data.

Prolonged absence from work often results in a lack of social structure and meaningful activity [20,43] and is associated with a reduced probability of eventual
return-to-work (RTW) and an increased probability of subsequent economic and social deprivation [43]. In the beginning of the sick leave episode the employee is often missed at work and employers will try to facilitate RTW. However, if sick leave continues, the employee will be replaced and the balance will be regained without the sick-listed employee and therefore RTW will become increasingly difficult to accomplish[44]. Thus, work itself can be an important factor in the RTW process[ref oost2]. It is also known that work is a significant contributor to the quality of life [44]. Long periods out of work lead to not only to a high risk on work disability, but also can contribute to 2-3 times increased risk of poor general health, 2-3 times increased risk of mental health problems and even 20% excess mortality.[46,19].

PREVALENCE
In the Netherlands, yearly 15.674 women receive a hysterectomy and 4.549 women a laparoscopic procedure for adnexal disorders.[41] These large number of operations has a large impact on absenteeism since it is estimated that approximately 57 percent of women, aged between 20-60 years, has paid work.[14]

HEALTH CARE PROBLEM/USUAL CARE
To reduce health care costs, there is an increasing trend to limit the duration of in-hospital care and to transfer postoperative care to outpatient care and primary care. However after discharge, care is given only on demand by gynaecologists and detailed recommendations on resumption of (work)activities are mostly not provided. Also general practitioners (GPs) frequently do not give advises about resumption of (work) activities. They often have too little knowledge and information about the physical demands of the (work) activities [3,47]. Occupational or insurance physicians are consulted only if patients have paid work and relatively late in the course of sick leave due to legislation. Often conflicting post-operative advices are given(see attachment table IC)[16,34] partly due to poor communication between medical doctors.[2,42]These factors may contribute to suboptimal peri-operative care. It is assumed that potential advantages of minimal invasive surgical procedures to accelerate recovery are not consumed.[21,10] A survey among 29 gynaecologists and OPs in the Netherlands confirmed the inconsistency in given recommendations after various types of hysterectomies. 25% of the gynaecologist never advice on work resumption and 37% sometimes. Specific instructions, related to type of job are never provided by gynaecologists versus only 43% of OPs.[21 The majority of patients of former observational studies confirmed these shortcomings in usual provided peri-operative care. National or international guidelines on this topic are lacking.

PATIENT COMPLIANCE & SATISFACTION
Due to this poor organization of health care, patients do not know whom to contact for support in case of postoperative complaints and how and when to resume which activity (see results focus groups). Because frequently conflicting advises are given, the patient’s compliance to advises is low. In addition, insufficient communication and lack of knowledge reduces convidence which may result in avoidance and late resumption of postoperative activities. The new peri-operative protocol is

developed to reduce patients uncertainties, irrational beliefs, delayed recovery, prolonged sick leave and to improve the quality of life(QOL).

COSTS FOR SOCIETY
Considering the high costs of sick leave and the adverse consequences of sick leave for employees in combination with the high number of gynaecological procedures per year in the Netherlands, prolonged sickleave induce unnecessary high costs for the society. Approximately 57% of the 20.000 women who receive a hysterectomy or a laparoscopic procedure for adnexal disorders have paid work. A study showed that mean sick leave exceeds 10 weeks.[21] It is obvious that more than 570.000 absence days yearly have a large impact on indirect costs for society. There is also strong evidence that long term sick leave contributes to higher consultation, medication consumption and hospital admission rates.[19,46]

Relevantie / Relevance

INNOVATION
In this project doctors and patients collaborate on a structured way in an innovative transmural care program which has been developed (ZonMw KKCZ 80-82000-98-610)to overcome shortcomings of current provided gynaecological peri-operative care, adjusted to patients needs. Patients needs were identified using focus groups. The transmural peri-operative care program contains a unique stepwise intervention: 1)a weblog, with detailed multidisciplinary recommendations on resumption of postoperative activities tailored to patients individual needs and physical demands at home and work, with tools for self-empowerment and with tools to improve communication with care-providers and employers. Followed by 2) a work-place intervention in case of prolonged sickleave to improve reintegration. The strong need for multidisciplinary recommendations for resumption of (work)activities for specific disorders was recommended by the Dutch Health Council[6], patients (focusgroups)and their representatives (see attachments).

MOTIVATION/K&E
The transmural care program will be included in a multidisciplinary light version guideline as a part of the KKCZ project and offered to organizations(NVOG, NHG, NVAB and NVVG)for eventual implementation in their official guidelines.
Cost-effectiveness of this care program has not been studied yet. In our opinion, a cost-effectiveness study should be performed before implementation of a new intervention in general practice, which is expected to occur after incorporation in guidelines. Official approval by all proffesional organizations is expected to occur in 2013. Acquired knowledge after implementation in guidelines will induce contamination of the “usual care group”, making future cost-effectiveness studies unreliable.

ICF
According to the HC, medical care by any physician regarding sick leave and work disability can and should be improved.[6]
The HC advises -in line with the ICF model of the WHO- that work participation should be a major treatment goal in multidisciplinary guidelines.[23] Work participation will be defined in this project in line with the definition in the ICF model: Participation will be related to paid work, but also to other types of role functioning, such as household chores, care for children or commuting.

POTENTIAL BENEFITS ON PATIENTS RECOVERY
- Improved recovery and accelerated resumption of (work)activities by 1) clear and consistent postoperative instruction on resumption of activities, improved self-empowerment, decreased confidence and 2) workplace intervention in case of prolonged sick-leave.
- Resumption of work contributes significantly to QOL and will prevent disability due to general and mental health problems and associated financial deprivation (Oosterom'09).
AD 1) clear instructions are able to reduce sickleave with 2-4 weeks after various surgical procedures (see systematic review part 1).
AD 2) workplace interventions are more effective in reducing sickleave (mean difference of 32 days with 95% CI 14,51)than usual provided care in case of low back pain or musculoskeletal disorders. Hazard ratio for time until lasting RTW was 1,7-2,0(see systematic review part 2).

EXPECTED EFFECT AMONG SPECIAL GROUPS
Since both communication problems and demanding jobs are more prevalent among ethnic and lower social economic groups, these groups are expected to benefit in particular from this transmural care program, in case they have access to internet. 90% of households had internet connection in 2009 in the Netherlands (Eurostat 2009) and 76% of low social classes had internet connection at home (Televisierapport 2009), and is increasing rapidly. Also internet access among elder people increases rapidly.

POTENTIAL ECONOMICAL BENEFITS
a) Reduced medical consultation due to improved care-coordination and communication.
b) Reduced costs due to less days with activity loss: yearly > 20.000 women receive a hysterectomy or laparoscopic adnexal surgery in the Netherlands (prismant 06). Based on studies(see systematic review part 1)[2-5], we estimate at least a reduction of 14 days for all procedures after the aplication of the intervention(=280.000 days/year). In addition 30% of patients had not resumed full work after 12 weeks (Dirkz). Workplace intervention in these patients is expected to improve RTW (see systematic review part 2) with 32 days (2.000 x 32= 64.000 days/year). Thus a total reduction of 344.000 to 408.000 days/year will be feasible for these procedures in the Netherlands.
c) Sickleave and associated health problems is strongly associated with increased medical consumption and compensation costs. This care program developed for gynaecological procedures may be applicable, after some adjustments, for other

surgical procedures. In the Netherlands we perform approximately 1.087.139 surgical procedures per year in general-, plastic- and cosmetic surgery, urology, orthopaedics and gynaecology[18].

Kennisoverdracht, implementatie, bestendiging / Knowledge transfer, Implementation Consolidation

C O N T E X T A N A L Y S I S

POLITICAL TRENDS
This project is in line with a number of political trends & advises of policy makers to improve the medical care for workers:
1. The Dutch Health Council adviced to improve medical care for sick listed patients and adviced to develop multidisciplinary guidelines.
2. CVZ, NVAB, LHV/NHG, BPV&W, CBO and the OMS initiated a number of projects to improve care for workers and cooperation between occupational and curative care.
3. It fits also in the policies of VUmc to enforce transmural care as well as collaboration between occupational and curative care.
4 Empowerment and reinforcement of primary care is increasingly implemented as a tool to reduce health care costs.

SCIENTIFIC TRENDS
This project fits in the current international and national scientific insights about multidisciplinary recommendations for resumption of (work) activities for sick listed patients. This project will contribute to the limited scientific evidence in this field. The recommendations will be in line with (inter)national guidelines to support health care providers advising patients about RTW.[46,24]

DECISION MAKERS
Professional organizations of gynaecologists,IPs,OPs and GPs as well as patients(NPCF) participated in an active way in this project. This will ensure an optimal knowledge transfer, implementation and adherence regarding the developed recommendations.

This project will add to the knowledge of IPs, OPs, GPs and gynaecologists about multidisciplinary recommendations for resumption of (work)activities after gynaecological surgery. The project can help them to better advice patients with work and their employers before and after surgery about resumption of activities and to improve the quality and outcomes of their care. It will reduce also conflicting advices. This project allows optimal benefits of new minimal invasive surgical techniques with respect to improved recovery and increased patients' satisfaction.

TARGET GROUPS: PATIENTS & EMPLOYERS
Patients as well as employers will benefit from multidisciplinary postoperative recommendations:
-They can lead to less conflicting advises and less patient uncertainties about resumption of activities.
-They can improve the quality of the care and outcomes for patients after gynaecological surgery.
-They can reduce sickness absence, productivity loss and costs due to wage replacement.
-Future target groups: They are relevant for patients undergoing all kind of surgical procedures.

K N O W L E D G E T R A N S F E R/ I M P L E M E N T A T I O N

Due to this project it is our ambition to accomplish the following implementation & knowledge transfer (KTE)objectives:
KTE-OBJECTIVE 1: INCORPORATION IN OFFICIAL GUIDELINES
The NVVG, NVAB, NHG and NVOG have been participate in the development of structured recommendations and innovative care program and underlined the need for the current K&E study of this care program. The developed guidelines will be send to these organizations to be used for future guideline development. We will encourage the organizations to implement this program only after coste-effectivess has been proven.

KTE-OBJECTIVE 2: INCORPORATION IN PATIENT INFORMATION LEAFLETS AND WEBSITES.
NPCF and professional organizations will participate in this project. For this reason these organizations will be asked to incorporate the developed recommendations into their relevant websites and patient information leaflets after proven Cost-effectiveness. The weblog will be available for patients after finishing the project.

KTE-OBJECTIVE 3: PRESENTATIONS
• By presentation of study results on at least 2 international congress, scientists and relevant health care professionals will be informed about the study results.
• By presentation at the annual congress of Dutch OPs 600 of the approximately 4220 occupational physicians will be potentially informed about the recommendations for postoperative resumption of (work) activities. Final goal is that 30% of the occupational physicians in the Netherlands are familiar with study results and/or content of the recommendations.
• By presentation at the 'Gynaecongres' almost all gynaecologists (1001) in the Netherlands will be informed about the study results. Final goal is that 50% of the gynaecologists in the Netherlands are familiar with recommendations for postoperative resumption of (work) activities.

KTE-OBJECTIVE 4: PUBLICATIONS
• Publication of the study results & recommendations in at least 3 articles in international peer-reviewed journals

• Publication in 2 relevant Dutch journals, e.g.: Tijdschrift voor Bedrijfs- en Verzek.-geneeskunde (oplage 3.600); Nederlands Tijdschrift Voor Geneeskunde (oplage 29.000); Medisch Contact (oplage 35.000); Ned. Tijdschrift voor Gynaecologie & Verloskunde (oplage 1600).
• This project will result in a PhD dissertation.

Doelstelling / Objective

O B J E C T I V E S

OPTIMISATION OF GYNAECOLOGICAL PERI-IOPERATIVE MEDICAL CARE
a. Providing uniform detailed peri-operative instructions to patients (based on transmural guidelines) and their care providers, for resumption of (work)activities. Provided recommendation/activity schedule will be tailored to patients activities at home and at work and by the individual interst of the patient.
b. Improving self-management(planning re-integration)and empowerment and reducing patients insecurity after surgery.
c. Improving communication between patient-doctor, patient-employer and between primary-secundary care (gynaecologist, GP and OP) to avoid conflicting advises and coordinate provided care (using ICT)
d. Early identification of patients at risk of delayed recovery and re-integration (using ICT weblog), followed by additional interventions (referral to specialists, instructions, and (ergonomic) work-adaptation).

RESEARCH QUESTIONS
1) What is the cost-effectiveness of this transmural care program compared to “usual care” in patients undergoing gynaecologic procedures?
2) What is the cost-utility of this transmural care program compared to “usual care” in patients undergoing a gynaecologic procedures?

SUBQUESTIONS:
- What is the effectiveness of this peri-operative care program on sick-leave, patients- and care-providers satisfaction
- What is the effectiveness of this care program on (recovery specific) QOL 2,6,8,12 and 36 weeks after surgery
- Which factors improve compliance
- Can RTW be predicted by prognostic factors and RS-QOL questionnaires?

GOALS
1) Compare the effect of both care programs on the quality of recovery, health related quality of life and resumption of(work)activities
3) Compare total costs of both care programs: programs itself, other health care consumption, of production loss and patients/ family costs
4) Comparing patients and doctors satisfaction and required time investments using the care programs
5) To develop a predictive model for RTW after gynaecological procedures
6) Knowledge transfer; papers in peer reviewed journals (3 international and 2 national), presentation at 2 national and 2 international congresses and writing PhD thesis. Implementation in official guidelines and daily practice after proven cost-effectiveness

Plan van Aanpak / Strategy

P R E V I O U S   S T U D I E S   T O   D E S I G N   T H E   S T U D Y

We performed several studies to develop an innovative transmural care program to improve the quality of peri-operative gynaecological care (STUDY 1-5; ZonMw 80-82000-98-610).
An innovative stepwise approach was used to develop evidence- and consensus-based recommendations and a patient Weblog, including a systematic review (STUDY 1) and a modified Delphi procedure among experts (STUDY 2). Focus groups were used to identify patients needs and beliefs regarding postoperative resumptions of work activities (STUDY 3). The used method was developed by the applicant under authority of College Zorg Verzekeringen (CVZ), Ned. Ver. Arbeids- en Bedrijfsgeneesk.(NVAB) and Orde van Medisch Specialisten (OMS).[28] According to the Dutch Health Council this innovative approach can bridge the gaps in existing evidence.[24] The developed transmural care program includes an interactive weblog, including tailored instructions for resumption of activities after the surgery, tools to improve communication and self-empowerments and a workplace intervention in case of recovery problems. The Weblog and instruction film were evaluated and approved by stakeholders and all patients in the focus groups (STUDY 4). The applicability, usefulness and effect of the innovative peri-operative gynaecological care program, including this weblog in combination with a workplace intervention in case of recovery and reintegration problems, is currently studied in a pilot study (STUDY 5). Note that most of the reported data in this grant proposal are not published yet, we would like to underline that this information is confidential.

S T U D Y 1   S Y S T E M A T I C   L I T E R A T U R E   R E V I E W
The systematic literature search focused on three interventions which are included in the transmural peri-operative care program (i.e. strict recommendations on return to work, workplace intervention and webbased intervention) on return to work.

Tree systematic literature searches and outcome will be discussed separately:
PART 1: Effect of convalescence instructions on return to work (RTW)

after gynaecological or abdominal surgery.
PART 2: Effect of workplace intervention on RTW in case of prolonged sick leave (a recent performed systematic cochrane review)[43].
PART 3: Effect of web-based interventions on patient empowerment (a very recent systematic review (in press) (Samoocha et al, JMIR)[37].

SYSTEMATIC REVIEW PART 1 : EFFECT OF CONVALESCENCE INSTRUCTIONS ON RETURN TO WORK AFTER GYNAECOLOGICAL OR ABDOMINAL SURGERY

DESCRIPTION OF THE STUDIED INTERVENTION
Standardized or well-defined peri-operative recommendations on resumption of (work)activities (convalescence recommendations) to patients with respect to gynaecological surgical procedures or abdominal surgical procedures.
OBJECTIVE
To determine the effectiveness of convalescence recommendations provided among patients undergoing gynaecological or abdominal surgery in preventing (long-term) sick-leave, when compared to usual care.
CRITERIA FOR CONSIDERING STUDIES FOR THIS REVIEW
a) Types of studies: all types of clinical studies, published as full papers in peer reviewed journals
b) Type of interventions: all kind of peri- or postoperative provided instructions, advice, counselling or recommendations on return to work, return to activities, resumption of activities or work activities
c) Type of patients: all patients undergoing gynaecological surgical procedures (in particular hysterectomy, prolaps and adnexal surgery) or abdominal surgery including bowel surgery and appendectomy for a benign indication
PRIMARY OUTCOMES ARE RETURN TO WORK (RTW) PARAMETERS
- Time until a lasting RTW: a period of absence from the first day of sick leave to full return to work in previous or equal work for at least four weeks without dropping out.
- Time until first return to work: a period of absence from wok because of sickness both preceded and followed by a period of at least one day at work
- Cumulative duration of sickness absence: total days of sick leave during the follow-up period.
SECONDAIRY OUTCOMES
Functional state, quality of life, general health, symptoms, pain, re-admission rate and costs
PERFORMED SEARCHES
Searches, combining all possible terms (see attachment) for items mentioned under b and c were performed in ARBO Bibliotheek Nederland, Cinahl, Cochrane Occupational Health Field Trials Register, Cochrane/CENTRAL and EMBASE, NIOSHTIC (occupational safety and health), PubMed (Medline) and PsylNFO. The reference list of relevant review articles and eligible studies were checked. Eligibility was assessed by two researchers JHU and TVN). In the second step full papers were studied in case the inclusion of exclusion could not be based on the first screening. Quality of the papers was evaluated using CONSORT criteria for clinical trials.
RESULTS SYSTEMATIC REVIEW PART 1
The combination of pre-, peri-, or post-operative and all possible terms of recommendations yielded without any limits yielded 14313 papers. The combination of this intervention with our primary outcome terms yielded 915 citizions. In combination with all possible terms for type of surgery applied to the patient as mentioned under c 215 citizions were retrieved. Applying the limits of “Clinical trial” and “Humans” 175 titles remained. Title screening yielded 25, and viewing the abstract After screeing the full papers, additional 8 were excluded, mainly because of additional provided interventions such as comparison of two types of surgical procedures (see flow-diagram). Characteristics of 7 included papers and assessed outcomes are presented in table IA and main results in table IB. None of the papers fulfilled all CONSORT criteria. Only 1 RCT compared the effect of defined convalescence recommendations compared to usual care provided after gynaecological surgical procedures. Our defined primary outcome parameter was not measured in this study and method of randomization was not reported. Duration of admission in the hospital was significantly shorter and given defined accelerated recovery instructions were cost-effective based on the calculated shorter admission. Cost-utility and Cost reduction due to changes in RTW were not measured. Quality of life measured with SF36 6 weeks after surgery were similar in both groups [18].
None of the studies measured the effect of provided defined convalescence instructions on lasting RTW, recurrence of sickness absence or cumulative duration of sickness absence. In total 3 studies could be identified, measuring first RTW after gynaecological surgical procedures in relation to provided convalescence recommendations. (see table IB). Results from one prospective cohort study compared to historic controls, indicated that the achieved first RTW could be shortened from median 6 weeks to 1 to 3 weeks after vaginal hysterectomy and prolaps-surgery [35]. In line with these data, accelerated first RTW was also reported after defined convalescence recommendations applied after inguinal hernia repair [1,12] and laparoscopic cholecystectomy [8]. A significant relation could be identified in a retrospective cohort study, studying the relation between provided RTW-recommendations and actual first RTW after abdominal hysterectomy, using a multilevel regression analyses. There was a large variation in provided recommendations, as a part of usual care in this study, in 17% of all cases no recommendations were provided at al. In the latter situation, first RTW was significant later with a median 122 (34-214) compared to the situations in which any recommendation was provided 89 (23-293) [16].
DATA ANALYSIS
Pooled analyses could not be performed.
CONCLUSIONS SYSTEMATIC REVIEW PART 1
Our review identied a few clinical trials studying the effect of convelescence recommendations, provided after gynaecological or abdominal surgery, on RTW. Well performed randomized controlled trials assessing the effect of convalescence recommendations as an independent intervention, provided with respect to gynaecological or abdominal surgical procedures, on RTW parameters are lacking. Prospective cohort studies indicate that well defined peri-operative provided convalescence recommendations are able to reduce sickness-related absence with a factor 2 or at least with 2 to 4 weeks compared to usual provided care. Studies using questionnaires among gynaecologists, surgeons, GPs and OPs report large variation in provided advice with respect to first RTW. In general, OPs and GPs advice a few weeks later to resume work compared to

gynaecologists or surgeons. An extensive part of care providers does not give any recommendation at all, varying from 17 to 25% [16,21,31]. Because of the low quality of evidence we found, the results should be interpreted with caution. Our systematic review underlines the need for a well designed RCT on this topic, including a sufficient long follow-up period.

SYSTEMATIC REVIEW PART 2: EFFECT OF WORKPLACE INTERVENTION ON RETURN TO WORK APPLIED IN PATIENTS SICK LISTED.
Results of a recent publishes Cochrane review of Oosterom et al will be published here.
We performed a very brief update until of the systematic literature search performed by Oosterom et al as published in a Cochrane systematic review 2009.[43]
The additional search update from 2007 to December 2009 yielded 11 papers in PsycINFO, 44 in Embase and 163 in CENTRAL. We could not identify any key paper after sreening the titles.
DESCRIPTION OF THE STUDIED INTERVENTION
The Cochrane Occupational Health Field has classified workplace interventions as appropriate for disability management.
In this review, workplace interventions are defined by either changes to the workplace or equipment, changes in work design and organization, changes in working conditions or work environment, and occupational (case) management with active stakeholder involvement of (at least) the worker and the employer [4]. Active involvement was defined as face-to-face conversations about return to work between (at least) the worker and the employer. Changes in the workplace and equipment include changes in the furniture or thematerials needed to performthe work. Changes in the work design and organization include changes in schedules or tasks, training in task performance, and altered working relationships with supervisor and co-workers.
The workplace interventions studied can be considered as a form of disability management for the individual worker that facilitates return to work by removing the barriers to return to work. Workplace interventions were compared with usual care (no intervention) or clinical interventions.
DESCRIPTION OF HOW THE WORKPLACE INTERVENTION MIGHT WORK
Evidence indicates that return to work is influenced by various psychosocial factors [39,48]. Long-term work disability is no longer seen simply as the consequence of a disorder but rather as the result of interactions between the worker and three systems: (1) the healthcare system, (2) the work environment, and (3) the financial compensation system [23,30]. Although this conceptual model has been derived from research on musculoskeletal disorders it can be applied to return to work processes for all types of work disability (including mental health problems and other health conditions) because both individual and work environment factors are involved and must be taken into account [44,49].
OBJECTIVE
1) To determine the effectiveness of workplace interventions in preventing long-term work disability among sick-listed workers, when compared to usual care.
CRITERIA FOR CONSIDERING STUDIES FOR THIS REVIEW
a Types of studies: all randomized controlled trials (RCTs) concerning workplace interventions aimed at preventing work disability by means of job accommodation or involvement of at least the worker and the employer, as key stakeholders, in the return to work process.
b Type of interventions: All kind of interventions focusing on changes in the workplace or equipment, work design and organization (including working relationships), working conditions or work environment, and occupational (case) management with active stakeholder involvement of (at least) the worker and the employer. These interventions comprise at least advice about changes in work processes to facilitate return to work or the preparation of a return to work plan with the worker and the supervisor.
c Type of participants: working age adults (18 to 65 years) who were on sick leave (full or part time). Workers with all types of work disability were included: musculoskeletal disorders, mental health problems, and other health conditions. The reasons for work disability could be either self-reported, diagnosed by a physician, or documented in an administrative file.
d Type of resources: ARBO Bibliotheek Nederland, Cinahl, Cochrane (Cochrane Occupational Health Field Trials Register, CENTRAL and EMBASE, NIOSHTIC (occupational safety and health),PubMed (Medline) and PsyINFO.
PRIMARY OUTCOMES
- Time until a lasting, Time until first return to work,
Cumulative duration of sickness absence.
SECONDAIRY OUTCOMES
Functional state, quality of life, general health, symptoms, pain, re-admission rate and costs
EXLUSION CRITERIA
Studies were excluded if the intervention was focused on primary prevention, not focused on return to work as the main goal, group based rather than individual based, focused on ’just’ education about ergonomics, not resulting in work adaptations, aimed at posture modifications only.
SELECTION OF STUDIES
Title and abstracts if available of all identified studies was studied by two review authors to check eligibility of the papers. In the second step full papers were studied in case the inclusion of exclusion could not be based on the first screening. Two authors independently extracted the data onto a pre-designed data extraction form and assessed the risk of bias of the RCTs based on an adapted version of the checklist recommended by the Cochrane Group. The criteria regarding blinding of intervention providers and participants were not used because the context of the workplace does not allow blinding.
RESULTS LITERATURE SEARCH AND STUDY SELECTION
The Cochrane literature search produced a yield 1350 titles in the original Cochrane search, of which 30 potentially eligible studies were selected based on the titles and or abstracts. After the examination of full publication papers, ten articles from six studies were left [5,6,9,29,45].
EFFECTS OF WORKPLACE INTERVENTION COMPARED TO USUAL CARE
The first pre-defined outcome regarding sickness absence, time until lasting RTW, significantly favoured the workplace intervention with a hazard ratio of 1.70 (95% CI 1.23 to 2.35) (figure 1A, attachment). The pooled analysis of the outcome on

time until first RTW showed that workplace interventions were more effective than usual care or clinical intervention pooled hazard ratio of 1.55 and 2.67, respectively (figure 2, attachement). Pooled analysis showed a significant advantage of the workplace interventions compared to usual care with a mean difference of 39.06 days (95% CI -61.86 to -16.26) (Figure 3). Pooled data on quality of life could not be performed. One study reported a significant difference between the two groups at 16-month follow up, in favour of the workplace intervention group.
Cost outcomes were not pooled as the outcomes were not considered comparable across the three studies . One study reported no significant difference in costs [5]. Two studies reported lower costs in the workplace intervention group [6,29].
CONCLUSION SYSTEMATIC REVIEW PART 2: WORKPLACE INTERVENTION
This systematic review identified six randomized controlled trials that evaluated the effects of workplace interventions on work disability due to various underlying disorders. All reported a beneficial effect on RTW outcomes with hazard ratio's varying between 1.6 to 2.7. compared to usual provided care or clinical interventions. Sickness absence could be reduced with a mean difference of almost 40 days.

SYSTEMATIC REVIEW PART 3: WEB-BASED PATIENT EMPOWERMENT
Effectiveness of web-based interventions on patient empowerment: a systematic review and meta-analysis.
This systematic review has been performed by Samoocha et al. and will be published in Medical Internet Research (in press JMIR)[37]. Therefore this information is highly confidential. A very brief summary of this search will be provided here.
OBJECTIVE
To evaluate if web-based interventions are effective in increasing patient empowerment compared to usual care or face-to-face interventions.
BACKGROUND
Patient empowerment refers to the enhanced ability of patients to actively understand and influence their own health status [11]. It focuses on control in individuals' experience of health, disease and illness, as well as the roles of health care organizations, communities and the broader health care system [37]. Since its introduction in health care in the 1970s, the popularity of the idea of patient empowerment has emerged in the context of several significant societal trends, such as a growth in health care consumerism and its resulting government's need for reduction in health care costs. Patient empowerment can potentially be used as a justification for cost-cutting as a part of transfer of responsibility for care to individual citizens. Furthermore, increased patient activism and organization led to more focus on patient empowerment initiatives. Revealingly, and in line of these tendencies, the World Health Organization (WHO) has described patient empowerment as a "prerequisite for health" and "a proactive partnership and patient self-care strategy to improve health outcomes and quality of life among the chronically ill".
METHODS
a) Type of studies: randomized controlled trials (RCT), quasi-randomized controlled trials, before-and-after studies and interrupted time series analyses were included in this review.
b) Type of patients: patients or clients with a medical problem were included in the review. Studies that included children and adolescents (<18 years old) were excluded in order to create more homogeneity in the study population.
c) Type of interventions: a web-based intervention, defined as all interactive web applications that are accessed via the Internet or an intranet. Studies including intervention that did not contain any aspects of health education or intention of health related behavioral change were excluded. Studies were only included if the control intervention consisted of either usual care or a face-to-face intervention.
d) Type of measures: empowerment or an empowerment related components. Given the absence of a generally accepted definition of empowerment, and conflicting views on how to measure empowerment, we decided to initially include all adjoining concepts often linked to empowerment. Examples of these are self-efficacy, mastery, self-control, self-esteem, perceived control, perceived competence or involvement in the decision making process.
SEARCHES
Systematic review by searching the MEDLINE, EMBASE and PsycINFO databases from January 1985 to January 2009 for relevant citations. From the 7096 unique citations retrieved from the search strategy, 14 randomized controlled trials (RCTs) met all inclusion criteria and were included (refs weergeven 1-14 samoocha). Pairs of review authors assessed the methodological quality of the obtained studies using the Downs and Black checklist. A meta-analysis was performed on studies that measured comparable outcomes. The GRADE approach was used to determine the level of evidence for each outcome.
RESULTS
In comparison with usual care or no care, web-based interventions had a significant positive effect on empowerment measured with the Diabetes Empowerment Scale (2 studies; SMD 0.61 [95% CI 0.29 to 0.94]), self-efficacy measured with disease-specific self-efficacy scales (9 studies; SMD 0.23 [95% CI 0.12 to 0.33]) and mastery measured with the Pearlin Mastery Scale (1 study; 2.95 [95% CI 1.66 to 4.24]). No effects were found for self-efficacy measured with general self-efficacy scales (3 studies; SMD 0.05 [95% CI -0.25 to 0.35]) and self esteem measured with the Rosenberg self-esteem scale (1 study; -0.38 [95% CI -2.45 to 1.69]). Furthermore, when comparing web-based interventions with face-to-face deliveries of the same interventions, no significant (beneficial or harmful) effects were found on mastery (1 study; 1.20 [95% CI -1.73 to 4.13]) and self esteem (1 study; -0.10 [95% CI -0.45 to 0.25]).
CONCLUSIONS
Web-based interventions showed positive effects on patient empowerment measured with the Diabetes Empowerment Scale, disease-specific self-efficacy scales and the Pearlin Mastery Scale. Because of the low quality of evidence we found, the results should be interpreted with caution.

CONCLUSION SYTEMATIC LITERATURE SEARCH PART 1, 2 AND 3:
There is a large variation in provided convalescence instruction and often very limited. Well defined peri-operative provided convalescence instructions after gynaecological surgical procedures may accelerate recovery and first RTW with 2 to 4 weeks. Workplace intervention provided in long-term sicklisted patients (with low back pain) reduces RTW measures (HR 1.6 to 2.7). Web-based interventions showed positive effects on patient empowerment measured compared to usual provided or no care.

2 DELPHI STUDY
Development of multidisciplinary recommendations for resumption of (work)activities after gynaecologic procedures, based on the literature review and structural consensus procedure among experts. A modified Delphi procedure was used to achieve consensus about the (graded) resumption of postoperative work activities among the expert panel. Recommendations are tailor made: differentiated by number of days/weeks after surgery and by surgical technique, and the underlying disorder. By repeated anonymous questionnaire rounds and group discussions it is possible to give feedback in a controlled way on the previous round to achieve in a short period of time a consensus opinion by a multidisciplinary panel of experts-doctors. It has been proven to be an efficient and useful method to achieve consensus in a multidisciplinary expert-group of medical doctors.[24] The expert team contained twelve representatives of professional organisations of gynaecologists, OPs and GPs (NVOG, NVAB, NHG), all completed the entire study:
ROUND 1: questionnaire
A systematic literature search was performed to assess reported convalescence after all types of hysterectomies and laparoscopic adnexal procedures in the same database sourches as used in systematic review part 1 and in additional databases reporting on mean RTW. A summary of the literature review was sent to all panel members to be used when completing the first Delphi questionnaire. In a first Delphi-questionnaire round relevant domains of functional limitation are explored by the panel. To determine the functional limitations we will used the Functionele Mogelijkheden Lijst (FML) i.e. functional limitations list (LISV, 2001)[13]. This instrument has been chosen because it has a legal status to assess functional limitations in the Netherlands and is used by all OPs, insurance physicians and labour experts in the Netherlands to assess and advise patients about functional limitations in work. For each case description above mentioned, the relevance of all FML-domains and the FML-items of each domain was scored by each panel member in the questionnaire separately, including a score of the uncertainty of their decision (on a 10-point scale: very uncertain to very certain). Finally the panel members were asked to score/rank in the questionnaire the obstacles for resumption of work activities derived from the literature.
ROUND 2: expert panel meeting
After the first Delphi round, for each item, the median values and range were calculated of their relevance scores (domains),of the limitation scores(items)and their certainty of each decision. These (anonymous) results were presented graphically (histograms) in a group meeting and discussed with the expert panel, providing the opportunity to explore on which domains and limitation items there was more or less consensus or large uncertainties among the panel members. Each item was discussed, after which the panel were asked individually to rate the limitation items again based on the group discussion and their own opinion. They had to rate the limitations with a maximum of certainty. A nominal group technique was used to reach consensus among the expert panel members about the limitation scores with the highest certainty score.
ROUND 3: draft recommendations to a select sample
Results of the second round were translated in draft recommendations about postoperative work activities (see attachment)including a list of medical and non medical obstacles for resumption of (work) activities. This draft was sent to a sample of doctors derived from the professional organisations which are represented by the panel members. 25 GP received the draft recommendations, 21 completed the questionnaires. OPs were requested by the website of the NVAB: 21 OPs initially responded, 19 completed the questionnaires. NVOG: 30 gynaecologists, 26 completed the questionnaire.
Total of 66 questionnaires of which 1 OP and 2 GP reported to be insufficiently familiar with the procedures to judge the guideline extensively, these were excluded.
63 responders, judged the presented results in the translated guideline, summary and graphically presented results of the 4 procedures. Major revisions were not requested and only minor revisions were proposed, these were presented to the experts and were part of the discussion during the last Delphi round. No major objections or obstructions against the consensus opinion regarding the recommendations for resumption of work activities were given.
ROUND 4: final expert panel meeting
The results of round 3 were presented and proposed minor revisions, by the aselect sample of representatives of the professional organisations as mentioned above, were presented and discussed. A nominal group technique was used again. Consensus was achieved in 100% of all activities after the discussed gynaecological procedures (illustrated by attachment), resulting in a final set of multidisciplinary recommendations and a list of obstacles for postoperative resumption of work activities, differentiated by surgical technique, disorder and time since surgery.
DEVELOPMENT OF THE FINAL CASE DESCRIPTION
Based on the final recommendations, we made several patient case descriptions of an uncomplicated course of (graded) resumption of activities for each surgical intervention (removal of uterus/ovaries) and surgical technique (abdominal /vaginal /laparoscopic),as a function of time since surgery (in days/weeks). These results were added as input to develop the patient weblog. Additional, a list of obstacles for resumption of work activities (co morbidities, complications, obstacles in work, private life etc,) based on the literature review and the modified delphi study were added to the case descriptions to identify causes for a deviant course of resumption of activities.

3 FOCUS GROUP STUDY
Focus group discussions with patients have been proven to be a very efficient and effective tool to identify their needs, attitudes and (illness) beliefs regarding postoperative recovery and resumption of work activities. Also potential obstacles, facilitators and relevant process indicators were discussed regarding the implementation of postoperative recommendations & case descriptions, exercises and (online) communication with doctors. Important critical success factors and characteristics of a weblog were discussed regarding the applicability, feasibility, compatibility, consumer friendliness, obstacles for usage of a weblog in practice.
Focus group discussions were all recorded, transformed into verbatim transcript and analysed.
Results: Patients who participated in the former observational study (see study 2) were divided in three groups based on their time to resume (work)activities: fast-, intermediate- and delayed recovery. We contacted at random 20 patients of each group to ask if they were interested to participate. The first responding 12 patients of each group were included in the focus groups.
Main reported short comings of current provided peri-operative care were: insufficient or no information on resumption of activities or work activities after the surgical procedure, inconsistency in given advise among gynaecologists, GPs and OPs.

Insufficient information provided on the surgical procedures. Insecurity in case of physical or mental postoperative complaints or delayed recovery, what to do and who to contact? Insufficient patient-doctor communication, insufficient guidance by OPs and difficulties with work-reintegration due to insufficient understanding of employer or required job adaptation. Possible interventions to overcome these problems using a weblog, possibilities to print summary of advised activity program to discuss with OPs and employers to plan re-integration already before the surgery and the possibility to receive additional help in case of reintegration difficulties or delayed recovery were judged very positive by the focus group members. With respect to the communication between gynaecologists and OPs focus group members had the opinion that the patient should be in the lead, thus direct contact between OPs and gynaecologists should not occur without approval of the patient. Besides these proposed interventions patients suggested a forum to communicate with other patients.

4 W E B S I T E E V A L U A T I O N B Y P A T I E N T S A N D S T A K E H O L D E R S

1. A demo-version of the weblog with post operative recommendations was developed by an ICT-specialist based on the results of the focus group discussions and the modified Delphi study among experts (see attachments). The weblog includes i) an interactive detailed schedule of resumption of activities tailored to patients needs and interests, dependent on usual activities performed at home and work and type of work, ii)an extensive list of frequently asked questions and iii)general information on the surgical procedures, iv) clear instructions on when and how to contact in case of problems or complications, v) an instruction film for patients and employers to illustrate common pitfalls during reintegration, vi)a forum to contact other patients and vii)alerting* method for physical-, mental- or work-related recovery problems and vii). *includes the RS-QOL(RI-10) measurement 2 weeks after surgery, which has very good abilities to predict prolonged(>8wks)sickleave (AUC-ROC was 0.88, 95%CI 0.74-1.03)[10].
2. This demo-version of the weblog was judged by 50 in stakeholders: all members of the focus groups, representatives of the patient- NCPF and professional organisations (NVOG, NVAB, NHG, NVVG) and members of the expert groups and 2 nurses and 2 gynaecologists of our department using a subset of questions on applicability, feasibility, compatibility, consumer friendliness, obstacles for usage in practice and completeness. Patients indicate their level of agreement with each items on a scale from 1 (completely disagree) to 5(completely agree). Results: This demo-version of the website, before final adjustments, was judged by the fast majority (92-100% agreed or fully agreed)as easy to use, with a clear and pleasant lay-out and was judged as very complete, no items discussed during the focus groups were missing, and that it contained only relevant information. Additional it was judged by 75 to 100% of the patients to provide very useful tools to improve communication with employer and other patients and 92% of the patients thought that they would use the website or recommend it to a friend in the current form.
3. Weblog was adapted by the ICT specialist based on the input provided by the patients and stakeholders, resulting in the final website (see illustrating attachments).
4 One of the shortcomings of current usual care mentioned by patients during the focus group study were insufficient guidance during reintegration, insufficient understanding by or cooperation with employers with respect to required job adaptations. We developed an instruction film to prevent these problems and improve responsibility of patients and employers by demonstrating common pitfalls during (work)reintegration (see attachment).

To view the website: http://www.ikherstel.nl/index.php (gebruikersnaam: ZonMW, wachtwoord 2009/31268)

5 P I L O T S T U D Y

We are currently performing a pilot study to study the applicability of the total care program. This care program includes 1)an interactive weblog with i) an interactive detailed schedule of resumption of activities tailored to patients needs and interest, ii)an extensive list of frequently asked questions and general information on the surgical procedures, iii) clear instructions on when and how to contact in case of problems or complications, iv) additional tools to optimize communication of the patient with doctors and employer, v) including an instruction film for patients and employers to improve reintegration, vi) alerting method for physical-, mental- or work-related recovery problems* and vii) a forum to contact other patients 2)workplace intervention in case of recovery problems, defined as resumption of full (work)activities later than 6 weeks after the surgical procedure, applied by a trained occupational specialist.
1. Research question pilot study:
The main research question for the pilot study is:
How is the innovative peri-operative care program, including patient weblog, postoperative recommendations for resumption of work activities and the workplace intervention, evaluated by patients, their employers and their health care professionals? (i.e. the applicability, compliance to, satisfaction, barriers etc.) Additionally, prognostic factors are studied and process evaluation is performed.
2. Recruitment of patients pilot study:
35 women, aged between 18-65 years, placed on a waiting list for a hysterectomy or laparoscopic adnexal surgery in one of the participating centers and fulfilling all inclusion criteria are asked to participate, consecutively. Exclusion criteria are: malignancy,(ectopic) pregnancy, deep infiltrating endometriosis, concomitant surgical procedures or major health problems affecting daily activities, sick listed for more than 6 months, dealing with a lawsuit to their employer, not able to understand or complete the questionnaires.
3. Measurement of process outcomes and process indicators in pilot study:
Time to resume (work)activities (measured by the weblog), a validated Recovery Specific Quality Of Life questionnaire RS-QOL (2,4, 6 and 12 weeks after surgery), number and duration of online patient-doctor communications, patient compliance to (recommendations and exercises in) the weblog (measured by the weblog itself) and direct and indirect costs (cost diaries).
-Patient satisfaction PSOHQ, patients' attitudes and opinions towards (usage of) the weblog, online communication with medical doctors, and workplace adaptation provided by OPs/OTs) if applicable; perceived conflicting advises regarding resumption of activities.
-Other stakeholders (gynaecologist, GP, OP, IP, employer) attitudes and opinions regarding the recommendations, use of the weblog and online communication and evt applied work-place adaptation.

Prognostic bio-psychosocial and work related factors are measured in the pilot study because they can influence the outcome and process indicators: Medical complications after surgery, pain score, Tampa, job content questionnaire (JCQ)and physical workload, measured with the Dutch Musculoskeletal Questionnaire(DMQ).
Follow-up data are not available yet. Inclusion started 2 months later as planned (website was adjusted according to the responses of stakeholders, in particular the interactive part took more time).
5. (If necessary,) the weblog will be adapted based on results of the pilot study to enhance the chances of implementation of the weblog on a broad scale.
After eventual adjustments and completion of the light-version guidelines it will be offered to professional organizations (NVOG, NVAB, NVVG, NHG) for incorporation in concerning guidelines and patient leaflets and websites. In general the organizations will adjust the guidelines according to own regulations, and mostly these procedures take about 2 years before final approval by all members of the individual professional organizations. We expect the guideline for example to be submitted to the gynaecongress meeting were guidelines should be presented in November 2012 after which final approval can occur at earliest during the next meeting of the NVOG members in May 2013, with earlies implementation in daily practice not before the end of 2013. However we will encourage the organizations to wait with implementation in their guidelines until cost-effectiveness has been proven.

C O S T - E F F E C T I V E N E S S S T U D Y

DESIGN
A multicentre prospective controlled trial, using a step-wedged design, comparing the innovative transmural perioperative care program, including workplace intervention (=intervention group) and usual given peri-operative care (=control group) in patients undergoing gynaecological surgery.

RECRUITMENT OF PATIENTS
Employed women (>8hrs/wk), aged 18-65 years, scheduled for a hysterectomy or laparoscopic adnexal surgery in one of the participating hospitals, will be asked to participate. Exclusion criteria: malignancy, pregnancy, deep infiltrating endometriosis, concomitant surgical procedures or major health problems affecting daily activities, sick listed for more than 6 months, dealing with a lawsuit to their employer, not able to understand or complete the questionnaires, no access to internet.

RANDOMIZATION
Randomisation will be performed at centre level. All centers will start with usual care during the first 2 months. Every 2 months one centre will implement the intervention and continue this untill the end of the study. The main advantage of this design is the prevention of possible contamination between provided innovative intervention and usual given care in the control group. As soon as doctors and nurses have been working with the new intervention, it will be difficult to return to usual care due to acquired insights.

As soon as they fulfill all inclusion criteria and have given informed consent, patients will be included in the study. Depending on the randomisation of the centre and period in which they are operated usual care or the transmural care program will be applied.

BLINDING
Patients, therapists and researchers cannot be blinded for the allocated treatment. The multidisciplinary team is not involved in assessing any of the outcomes. The analyses of the data by the researcher will be blinded.

INTERVENTIONS

CONTROL GROUP: usual care
Detailed peri-operative instructions concerning resumption of (work)activities are mostly not provided. In this study, patients receive an access code to a basic website with general information. After discharge, secondary medical care in only given on demand, except for one outpatient visit six weeks after surgery in some clinics. OPs are usually only involved in postoperative care in case sick leave exceeds 6 weeks, in the framework of the Gatekeeper Law.

INTERVENTION GROUP: multidisciplinary peri-operative care program including an interactive weblog and additional workplace intervention in case of delayed recovery:
- Detailed uniform recommendations on resumption of activities, provided prior to surgery, on postoperative physical activities tailored to personal situation and type of work, allowing planning of (work)activities.(see attachment). After the surgery this plan will be adjusted in case of complications (see attachment).
- The website provides tools to decrease insecurities: a)extensive list of frequently asked questions and possibility to ask additional questions, b)general information on the surgical procedure(s), c)extensive list with explanations of often used medical terms, d)clear instructions when, who and how to contact various care providers, patient forum to contact other patients.
- The website provides tools for patients and their care-providers and employers for optimal communication and work-reintegration after surgery, including a film illustrating common pitfalls during reintegration (see attachment).
- The weblog provides an alerting system of any physical, mental- or work-related recovery problems. This includes the measurement of RS-QOL (RI-10)2 weeks after surgery, which has proven to predict prolonged(>8wks)sick-leave (AUC-ROC was 0.88, 95%CI 0.74-1.03)[10].
2) In case of recovery problems, i.e. if ful returned to work is not expected to occur 6 weeks after the surgery, additional stepwise workplace intervention is offered by a trained occupational therapist(OT).

WORKPLACE INTERVENTION
The workplace intervention consists of work(place) adaptations and is based on active participation and strong commitment of both the patient and employer. The workplace intervention is based on methods used in 'participatory ergonomics' [43,44]. Prior to the start of the study, three occupational therapists (OT)trained by an expert will provide the workplace intervention protocol. It consists of:
1 Patient's workplace observation and inventory and ranking patient's tasks and obstacles for RTW by the patient.
2 Inventory and ranking patient's tasks and obstacles for RTW by the patient's supervisor
3 Patient, patient's supervisor and the OT brainstorm and discuss about as many solutions as possible to clear the obstacles for RTW.
The aim of the workplace intervention is to achieve consensus between patient and supervisor regarding feasible solutions for the obstacles for RTW. The solutions are judged on availability, feasibility and solving capability. After consensus, the OT, patient, patient's supervisor and potential other stakeholders agree on a plan of action.
This intervention has proven to accelerate resumption of daily- and work activities with 4-6 weeks and was cost-effective in patients with lower back pain. Compliance to this protocol was high and satisfaction was good. (see systematic review part 2).

CONTAMINATION
As randomisation is performed at site level, using the step-wedged design, contamination between usual care an intervention is very low. We will ask concerning organisations to implement the new care program after it has been proven to be Cost-effective, thus after the end of the K&E-study.
However, despite the step-wedged design, during the transition period from usual care to intervention at each site, a few patients in the usual care group and patients in the intervention group may be treated at the same moment by the same gynaecologists, however the role of the gynaecologist will be minor given the fact that instructions on resumptions of activity will mainly be performed by the weblog and by a specialised OTs. The latter will only treat patients in treatment group. In case both type of patients are admitted at the same time in one site, we will offer these patients different rooms and nursing by different nurses.

EFFECT EVALUATION
Our intervention has 2 main goals:
1)improvement of quality of recovery
2)acceleration of resumption of (work)activities
Return to work should be considered as an outcome measure reflecting functional and societal participation. The Health Council advises, in line with the ICF model of the WHO, that work participation should be a major treatment goal in multidisciplinary guidelines. RTW was also the primary outcome measure in our former approved ZonMW project(80-82000-98-610). We consider both goals as equal important.

PRIMARY OUTCOMES
The primary outcome measures in this study are therefore
1) a validated (27) Recovery Specific Quality Of Life questionnaire: RS-QOL(RI10) 2, 4, 6 and 12 weeks after surgery.
2)Return to work (RTW): time to full resumption of normal (work)activities: defined as: duration of sick-leave with CMDs in calendar days from the day of surgery until full RTW in own or other work with equal earnings, for at least 4 weeks without (partial or full)recurrence, as registered by occupational health services. This means that recurrences of sick leave within 4 weeks of full RTW are considered as belonging to the preceding period of sick leave.

SECONDARY OUTCOMES:
Secondary outcomes are total number of days of sick leave during the follow-up, Quality of life, coping style, job content, and attitude, social influence, and self-efficacy determinants(Pearlin Mastry Scale)and satisfaction with provide care-program. Cost-effectiveness will be evaluated from the societal perspective. A process evaluation will also be conducted.

PROCESS EVALUATION
A brief process evaluation is conducted for all patients included with respect to actually received recommendations, their attitude and their complience to these recommendations. Employee satisfaction is measured with the short version of the Patient Satisfaction with Occupational Health Services Questionnaire (PSOHQ). Additional, process evaluation of the workplace intervention will conducted for the first 35 patients receiving the actual workplace intervention. A questionnaire will be sent to their supervisor, the OP and the RTW coordinator. For employees, the questionnaire is included in the questionnaire after 3 months and contains questions about employee satisfaction, the work adaptations chosen, the expected effect of work adaptations, and the compliance with workplace intervention process. In addition, the barriers for RTW, the solutions and the RTW plan discussed in the meetings are collected within standardized schemes. All identified barriers for RTW and solutions will be analyzed qualitatively and classified by two researchers independently. The classification will be based on a simplified version of the ‘Ergonomic Abstracts’ classification scheme.

ECONOMIC EVALUATION
Direct costs of health care usage are measured by the Tic-P questionnaire. The Tic-P is developed for medical costs relevant to the treatment, such as visits to general practitioner, occupational physician, gynaecologist, admission into a hospital, use of medication etc. Health care costs will be valued according to the prices suggested in the guidelines for economic evaluation in The Netherlands. If cost-guidelines are not available, costs will be estimated using real prices or population-based estimates if available in the literature. Costs of lost productivity caused by (partial) sick leave due to CMDs are calculated from the number of days of sick leave and lost earnings, as provided by the occupational health services. Indirect costs can be calculated using the friction cost approach and the human capital approach, based on income as provided by the employee or as derived from function and age. To compare the results of the cost effectiveness analysis with other conditions, general health status is

measured according to the standard Dutch version of the EuroQol EQ-5D.

MEASUREMENTS
This study has a one-year follow-up with measurements scheduled at baseline, 2 6 and 12 weeks, 6 and 12
months after surgery. Data on absenteeism are registered continuously by the occupational health services and will be acquired from the registration systems after the one-year follow-up. These data will be checked with self-reported information on the weblog. If the data are not consistent, the OP will be asked for clarification.
- RTW: Total days of self reported sick-leave during follow-up (measured by weblog).
- QOL:(SF36, euroQOL) before, and 12 and 36 weeks after surgery
- PSOHQ and patients and care-providers satisfaction with care-program (weblog, communication, workplace intervention 12 and 36 weeks after surgery
- The Job Content Questionnaire(JCQ)is used to measure job content at baseline and 3 months. Job content data can either be prognostic or provide insight in working mechanism of the workplace intervention
- PROGNOSTIC MEASURES: Sick leave in the past year, medical complications, pain score, Tampa and Dutch Musculoskeletal Questionnaire(DMQ)
- COST DATA: monthly questionnaire to assess direct and indirect costs/costs diary/Tic-P
- PROCES INDICATORS: Pearlin Mastry Scale, patient compliance to (recommendations and exercises in) the weblog (measured by the weblog itself), patients' attitudes and opinions towards (usage of)the weblog and communication with medical doctors, Other care providers(gynaecologist, GP, OP, IP, employer) attitudes and opinions regarding the recommendations, use of the weblog.

STATISTICAL ANALYSES
Regression analysis (analyses of covariance) with the outcome measure at follow-up as the dependent variable, adjusted for the outcome measure assessed at baseline if appropriate, will be used to assess effectiveness. Potential confounders are prognostic dissimilarities. The central independent variable is the treatment arm (intervention or usual care) to which the patient is allocated.
Survival analysis will be used to analyse sick leave data with regard to the first period of sick leave. To describe the sick leave duration until lasting RTW in both groups,
the Kaplan Meier method will be used. The Cox proportional hazard model will be applied to calculate hazard ratios.
Differences in total days of sick leave during the year of follow-up will be analysed by using the Student’s T-test.
RI-10 outcomes will be analysed using generalised estimating equation (GEE). Statistical analyses will be performed at the individual level and according to the intention-to-treat principle, which will be compared to the per-protocol analyses.
Additionally, extra costs of the care program will be evaluated.

POWER ANALYSES
Power calculation was performed on RTW, since the number of needed patients to achieve sufficient power is much larger for RTW, using survival analyses than for RI-10, using GEE analyses.
We expect a Hazard Ratio of 2.0 for Return to work based on the results of a Danish study using strict advices on resumption of activities after gynaecological surgery and on the reported results of a workplace intervention (Ottesen'03, see systematic review part 2). To achieve a power of 0,8, with an alpha of 0.05, considering a HR of 2.0, , using survival analyses and considering a 10% drop-out rate, 74 patients are needed. Applying a correction for a step-wedged (balanced) design with 7 centers and 8 time-events and considering an intraclass correlation coefficient of 0.05, 280 patients should be included.

COST-EFFECTIVENESS ANALYSES
Direct, indirect and total costs will be computed for each patient. Bootstrapping will be used for pair-wise comparison of the mean groups to calculate mean
differences in direct, indirect and total costs between the two groups. Confidence intervals (95%) will be obtained by bias corrected and accelerated bootstrapping. To assess the cost-effectiveness ratios of the intervention, the difference in mean costs between the groups will be divided by the difference in RTW between the groups. These ratios will be graphically presented in a costeffectiveness plane. Acceptability curves will also be presented. Similarly, utility assessed with the EuroQol EQ-5D will be used to estimate the incremental costs per QALY gained in a cost-utility analysis.

SPECIAL GROUPS
In the current study only women will be included, however when applied in the future for other types of surgery, both women and men will be included. Given the fact that both communication problems and physical demanding jobs are more prevalent among ethnic and lower social economic groups, in particular these groups are expected to benefit from this innovative multidisciplinary care program. Therefore these patients will also be included in the study. Given the fact that in the Netherlands, 90% of households have internet access and broadband internet connections(Eurostat 2009), and 78% of lower social classes it is expected that the majority of the patients will be able to participate. Social class, ethnicity and age will be taken into account in the final multi-level analyses. In the current study we do not include patients aged below 18 or above 65 years because one of our main endpoints is return to work. However internet access is accelating in among elder people and in particular this group seems to benefit from well defined accelerating rehabilitation program after surgical procedures [38]. It is expected that in the future, after implementation in daily practice, this group will benefit from the intervention applied in this protocol.

FEASIBILITY
The feasibility of the study is excellent. the research group has extensive experience with this type of studies and proposed intervention. Seven hospitals in the area of Amsterdam approved to participate in this study (VUmc, SLAZH, OLVG, Kennemer Gasthuis, Spaarne hospital, Amstelveen and Almere hospital). They account for 799 hysterectomies and 621 laparoscopic adnex surgeries, for a benign disease (excluding prolaps and ectopics pregnancies) each year. Assuming that 50% of these do

not meet any exclusion criteria and are willing to participate, 710 patients are eligible each year. Thus a recruitment of 280 patients in a period of 16 months is highly feasible. This means that every centre should include 5 patients every 2 months.

PLANNING: January 2011- Dec 2014 (see attachment)

1 Innitiation phase (6 months; Jan 2011 - June 2011)
- protocol completion
- optimisation weblog and database
- final approval METC's
- preparations logistics participating centers

2 Recruitment and inclusion phase (16 months; July 2011- Nov 2012)
- 280 patients, every 2 months one centre will implement the intervention, before that period usual care will be provided in that centre:
  2 months: 7 centres aplly usual care
  2 months: 6 centres usual care and 1 intervention
  2 months: 5 centres usual care and 2 intervention
  2 months: 4 centres usual care and 3 intervention
  2 months: 3 centres usual care and 4 intervention
  2 months: 2 centres usual care and 5 intervention
  2 months: 1 centre usual care and 6 intervention
  2 months: 7 centres apply the intervention

3 Follow-up phase (26 months; Aug 2011 - Dec 2013)
- It starts as soon as the first patient had the surgical procedure and ends 12 months after the last patient has been operated
- Data collection on absenteeism from occupational health services registrations

4 Analytic phase (9 months; June 2013 to February 2014)
- data-analysis

5 Knowledge transfer and Publication phase (10 months; March 2014 - December 2014
- writing 2 Dutch and 3 international papers
- performing presentations at 2 Dutch and 2 international congresses
- writing PhD-thesis

PATIENTS AND STAKEHOLDERS PARTICIPATION
Focus groups were used to identify patients needs and beliefs regarding postoperative work resumption, which were taken into account during the development of the weblog. Additional, patients representatives (NPCF (Ned Patienten en Consumenten Fed.)and Welder) participated also in the development of the patient weblog. This weblog supports patients in an interactive way to resume work
according the recommendations/graded activity program and to identify any recovery problem. In addition, this weblog facilitates communication between patients, the medical specialist and primary care physicians and will monitor the resumption of activities during recovery. Adjustments of the weblog were made based on the evaluation of a demo-version by patient's representatives and patients participating in the focus groups.

USERS
Besides the patients also gynaecologists, GPs, OPs, and insurance physicians (IP) will use the website. Representatives of the official organizations (NVOG, NHG, NVAB, NVVG) participated in the development and approval of the weblog, the uniform instructions on resumption of (work)activities after gynaecological surgery. In the pilot-study all users, including patients, will be asked to evaluate the usefulness, applicability of and satisfaction with the peri-operative care program. The weblog also includes process indicators to measure patients compliance.
After completion of the study, final recommendations will be formulated and sent to these organisations to be used for guideline development.

TARGET GROUPS: PATIENTS & EMPLOYERS
Patients as well as employers will benefit from multidisciplinary postoperative recommendations:
-They can lead to less conflicting advises and less patient uncertainties about resumption of activities.
-They can improve the quality of the care and outcomes for patients after gynaecological surgery.
-They can reduce sickness absence, productivity loss and costs due to wage replacement.
-Future target groups: They are relevant for patients undergoing all kind of surgical procedures.

Expertise, voorgaande activiteiten en producten / Expertise, prior activities and products

Prof. dr. J.R. ANEMA is both an occupational physician and an insurance physician. Since 2004 he is
employed as assistant professor and since 2010 as professor at the Department of Public and Occupational Health of the VU University Medical Center and the EMGO Institute in Amsterdam. Since 2005 he is also part time
employed at the Research Center for Insurance Medicine AMC UWV VUmc. He was formerly employed
as a senior researcher at TNO Work & Employment(1999-2004).

His main research topics are: 1. (Cost-)effectiveness of work disability prevention interventions 2. Improvement of cooperation between curative and occupational healthcare. He is/was project leader of several national projects. In many projects he collaborated with other academic research institutes in Amsterdam, Rotterdam, Nijmegen and Groningen and Dutch Medical Associations of GPs(LHV), OPs (NVAB), IPs (NVVG) and medical Specialists (OMS). He is/was involved in the development of a number of guidelines for the NHG/LHV, the Health Council and NVAB. He is member of the authorisation committee of the NVAB-guidelines. He is currently supervising many PhD-students.
He obtained in 1999 a personal governmental NWO-AGIKO-grant for his PhD-project. In 2002 he received the Zielhuis award of the Dutch Medical Board for Occupational Medicine(NVAB). In 2005 he has been awarded by the Netherlands Organisation for Health Research and Development (ZonMw) as ‘ZonMw Clinical Fellow 2005-2010' to develop a research line on 'Transmural occupational care’.
He has many international scientific collaborations. He is assigned in Canada since 2003 as a lecturer, since 2005 as a mentor and since 2008 as a member of the Programme Executive Committee for the CIHR strategic training program Work disability prevention. He is part of an international publishing network on work disability prevention. Since 2007 he is member of the editorial board of the Journal of Occupational Rehabilitation and Tijdschrift voor Bedrijfs- en Verzekeringsgeneeskunde.

Dr. J.A.F. HUIRNE is working as a gynaecologist on the Vumc and is specialized in minimal invasive surgical techniques. She attained a visiting scholarship in endoscopy in 2007 (fully paid) on the University hospital in Oslo in Norway. She is actively involved in the training of residents in Obstetrics and Gynaecology. After obtaining her PhD degree (cum laude) in February 2007, she is coaching four PhD-students and is participating in several national and international research projects. She is invited for lectures on several national and international congresses. She wrote several bookchapters and has an extended list of publications and presentations and is reviewer of several international journals. She is secretary of the Minimal invasive committee of the VUmc and member of the Dutch society of gynaecological endoscopy, Dutch society of endoscopic Surgery, European society of gynaecological endoscopy and European society of Human Reproduction.

Prof. dr. H.A.M. BRÖLMANN, is head of the Department of Gynaecology/oncology of the VUmc in Amsterdam. His professorial chair is in benign gynaecology and in particular endoscopic surgery. He supervised several AIO’s towards their thesis in this field. Several papers and presentations of his students were awarded on international congresses. He has an extended publication list in international and national peer reviewed papers. He developed three national gynaecological guidelines and wrote more than thirty chapters in national and international gynaecological books. He is collaborating with and is invited for lectures by several international research groups. He is
editor and of the journal ‘Gynecological Surgery’. He is founder of the Dutch society of gynaecological endoscopy. He is vice-president of the European society of gynecological endoscopy. He is actively involved in training of residents and gynaecologists as aFaculty member of the national council for resident training Obstetrics and gynaecology and of the Eindhoven and Amsterdam laparoscopic training centre.

Prof. dr. M.W. VAN TULDER is a professor of Health Technology Assessment at the Department of Health Sciences of the VU University in Amsterdam. He has ample expertise in economic evaluations alongside randomized controlled trials in primary care. He has supervised many economic evaluations funded by ZONMW programmes Health Care Efficiency Research (Doelmatigheidsonderzoek) and Prevention and has published more than 190 articles in international scientific journals.

Publicaties / Publications

•ANEMA JR, Van Der Giezen AM. [Little communication between company doctors and treating physicians with respect to hindrances to return to work after prolonged absence from work because of low back pain]. Ned Tijdschr Geneesk 1999;143:572-5.

•ANEMA JR, et al. Ineffective disability management by doctors is an obstacle for return-to-work: a cohort study on low back pain patients sicklisted for 3-4 months. Occup Environ Med. 2002;59:729-33.

•ANEMA JR et al. Participatory Ergonomics as a Return-to-Work Intervention; A future challenge? Am J Ind Med 2003; 44 273-81.

•Steenstra IA, ANEMA JR, Bongers PM, Vet HCW de, MECHELEN W van. Cost effectiveness of a multi-stage return to work program for workers on sickleave due to low back pain. Design of a trial. BMC Musculoskelet Disord. 2003 Nov 21;4(1):26.

•ANEMA JR et al. The effectiveness of ergonomic interventions on return-to-work after low back pain: a prospective 2-year cohort study in 6 countries on low back pain patients sicklisted 3-4 months. Occup Environ Med Apr 2004;61(4):289-94.

•Weevers HJA, Van der Beek AJ, ANEMA JR, Van der Wal G and Van MECHELEN W. Work-related disease in general practice: a systematic review. Family Practice 2005; 22: 197–204.

•Young AE, Wasiak R, Roessler R, McPherson KM, ANEMA JR, van Poppel MNM. Return to work following work disability: Stakeholder motivations, interests and concerns. J Occup Rehabil. 2005;15:543-56.

•Young AE, Roessler R, Wasiak R, McPherson KM, van Poppel MNM, ANEMA JR. A developmental conceptualization of

return to work. J Occup Rehabil. 2005;15:557-68.

•Buijs PC, van Dijk FJ, Evers M, vd Klink JJ, ANEMA H. Managing work-related psychological complaints by general practitioners, in coordination with occupational physicians: a pilot study. Ind Health. 2007;45:37-43.

•ANEMA JR, et al. Multidisciplinary rehabilitation for subacute low back pain: graded activity or workplace intervention or both?: a randomized controlled trial. Spine. 2007;32:291-8.

•van Oostrom SH, ANEMA JR, et al. Development of a workplace intervention for sick-listed employees
with stress-related mental disorders: Intervention Mapping as a useful tool. BMC Health Serv Res. 2007;7(1):127.

•Wasiak, R, Young, AE, Roessler, RT, McPherson, KM, van Poppel, MNM, ANEMA, JR. Measuring return to work. J Occup Rehabil. 2007;17:766-81. Epub 2007 Oct 11. Review.

•Kremer AM, ANEMA JR. Duration of recovery in specific medical conditions. A joined framework for OPs and clinical specialists. CVZ/TNO-rapport 018.10275.01.01, Hoofddorp, 2004.

•ANEMA JR, et al. A step forward, but a long way to go. Collaboration by occupational physicians and general practitioners in 10 regions in the Netherlands. TNO rapp.nr. 14372, Hoofddorp, 2003.

•ANEMA JR, et al. Collaboration by occupational physicians and general practitioners. Guidelines for practice. Medisch Contact 2001;20: 790-93;

•Venema A, ANEMA JR, et al. Samenwerking tussen huis- en bedrijfsartsen. Longitudinaal onderzoek naar aanleiding van regionale samenwerkingsprojecten. Tijdschrift voor gezondheidswetenschappen 2005;83:286-290.

•Kremer AM Buijs PC, van den Berg R, de Vroome E, van Dijk FJ, ANEMA JR, van Heesen BJ. Ontwikkeling richtlijn arbeidsanamnese voor medisch specialisten. Hoofddorp, 2005 TNO rapport 018.10281.01.01

•Amstel RJ ANEMA JR et al. Leidt een inadequate begeleiding tot langer verzuim? Hoofddorp, 2005
TNO rapport 10037

•Buijs P en ANEMA H. LHV/NVAB-Leidraad voor huisarts en bedrijfsarts bij de sociaal-medische begeleiding van arbeidsverzuim. Handboek Bedrijfsgezondheidszorg D5-1/1-D5/1/39.

•Samoocha D, Bruinvels DJ, ANEMA JR, et al. Empowerment of disability benefit claimants through an interactive website: design of a randomized controlled trial. BMC Medical Informatics and Decision Making 2009, 9:23

•Steenstra IA, Knol DL, Bongers PM, ANEMA JR et al. What works best for whom? An exploratory subgroup analysis in a randomized controlled trial on the effectiveness of a workplace intervention in low back pain patients on return to work. Spine 2009;34:1243-9.

•Lambeek LC, van Mechelen W, Buijs PC, Loisel P, Anema JR. An integrated care program to prevent work disability due to chronic low back pain: A process evaluation within a randomized controlled trial. BMC Musculoskelet Disord.;10:147

•de Jong T, Heinrich J, Blatter BM, ANEMA JR, van der Beek AJ. The feasibility of a web-based counselling program for occupational physicians and employees on sick leave due to back or neck pain. BMC Med Inform Decis Mak. 2009 Nov 6;9.46

• Lambeek LC, van MECHELEN W, Knol DL, Loisel P, ANEMA JR. Integrated care for chronic back pain: an RCT evaluating a systems approach to reduce disability in working and private life. In press BMJ.

•BRÖLMANN HAM, Vonk-Noordgraaf A, Bruinvels D, de Vet HC, Dirksz A, HUIRNE JAF. Can prolonged sick leave after gynecological surgery be predicted? An observational study in the Netherlands. Surgical Endoscopy 2009, jan 1.

•HAM BRÖLMANN, MY Bongers. Resultaten van laparoscopisch geassisteerde vaginale hysterectomieen, 1993-1997. Ned T Geneesk 98;142:2431

•Kluivers KB, Hendriks JC, Mol BW, Bongers MY, Bremer GL, de Vet HC, Vierhout ME, BRÖLMANN HA. Quality of life and surgical outcome after total laparoscopic hysterectomy versus total abdominal hysterectomy for benign disease: a randomized, controlled trial. J Minim Invasive Gynecol.
2007;14:145-52.

•HUIRNE JA et al. What is the impact of surgical expertise and how to get it? Gynaecological surgery 2008 5:265–267.

•Kluivers KB, Mol BW, Bremer GL, BRÖLMANN HA, Vierhout ME, Bongers MY. Pelvic organ function in randomized patients undergoing laparoscopic or abdominal hysterectomy. J Minim.Invasive.Gynecol 2007;14:442-48.

•BRÖLMANN HAM et al. Levels of evidence in endoscopic research: a longitudinal survey. Gynecol Surgery 2004;1: 91-95.

•BRÖLMANN HAM et al. A randomised comparison and economic evaluation in laparoscopic assisted hysterectomy and

abdomminal hysterectomy. Brit J Obstet Gynaecol 2002;109:1427-28.

•BRÖLMANN HAM, et al. Declining trend in major gynaecological surgery in The Netherlands during 1991-1998. Is there an impact on surgical skills and innovative ability? Brit J Obstet Gynaecol 2001;108:743-748

•Rebergen DS, Bruinvels DJ, van TULDER MW et al. Cost-effectiveness of guideline-based care for workers with mental health problems. J Occup Environ Med 2009;51:313-22

•Bosmans J, de Bruijne M, van Hout H, van Marwijk H, Beekman A, Bouter L, Stalman W, van TULDER M. Cost-effectiveness of a disease management program for major depression in elderly primary care patients. J Gen Intern Med. 2006 Oct;21:1020-6

•Bosmans JE, Brook OH, van Hout HP, de Bruijne MC, Nieuwenhuyse H, Bouter LM, Stalman WA, van TULDER MW. Cost effectiveness of a pharmacy-based coaching programme to improve adherence to antidepressants. Pharmacoeconomics. 2007;25:25-37

•Bosmans JE, Hermens ML, de Bruijne MC, van Hout HP, Terluin B, Bouter LM, van TULDER MW. Cost-effectiveness of usual general practitioner care with or without antidepressant medication for patients with minor or mild-major depression. J Affect Disord. 2008 Nov;111:106-12

•Van der Roer N, van TULDER M, van MECHELEN W, de Vet H. Economic evaluation of an intensive group training protocol compared with usual care physiotherapy in patients with chronic low back pain. Spine 2008;33:445-51

Referenties / References

(1) Ambach R, et al. Back to work more quickly after an inguinal hernia repair. Mil Med 2000;165:747-750.
(2) Anema JR, et al. Little communication between company doctors and private physicians with respect to hindrances to return to work after prolonged absence from work because of low back pain. Ned Tijdschr Geneeskd 1999 13;143:572-575.
(3) Anema JR, …, Van Mechelen W. Ineffective disability management by doctors is an obstacle for return-to-work: a cohort study on low back pain patients sicklisted for 3-4 months. Occup Environ Med 2002 ;59:729-733.
(4) Anema JR. Low back pain, workplace intervention and return-to-work. Thesis, discussion; 2004.
(5) Anema JR, et al. Multidisciplinary rehabilitation for subacute low back pain: graded activity or workplace intervention or both? A randomized controlled trial. Spine 2007;32:291-298.
(6) Arnetz BB, et al. Early workplace intervention for employees with musculoskeletal-related absenteeism: a prospective controlled intervention study. J Occup Environ Med 2003;45:499-506.
(7) Bensing J. Doctor-patient communication and the quality of care. Soc Sci Med 1991;32:1301-1310.
(8) Bisgaard T, et al. Factors determining convalescence after uncomplicated laparoscopic cholecystectomy. Arch Surg 2001 Aug;136:917-921.
(9) Blonk RWB, et al. Return to work: A comparison of two cognitive behavioural interventions in cases of work-related psychological complaints among the self-employed. Work & Strass 20[2], 129-144. 2006.
(10) Brolmann HA, …, Huirne JA. Can prolonged sick leave after gynecologic surgery be predicted? An observational study in the Netherlands. Surg Endosc 2009 Jan 1.
(11) Bruegel RB. Patient empowerment--a trend that matters. J AHIMA 1998 Sep;69(8):30-33.
(12) Callesen T, et al. Short convalescence after inguinal herniorrhaphy with standardised recommendations: duration and reasons for delayed return to work. Eur J Surg 1999 Mar;165(3):236-241.
(13) CBBS. Functionele Mogelijkheiden Lijst, UWV, Amsterdam. 2002.
(14) Centraal Bureau voor de Statistiek. Statline databank. 2008.
(15) Cheraghi-Sohi S, et al. Can the feedback of patient assessments, brief training, or their combination, improve the interpersonal skills of primary care physicians? A systematic review. BMC Health Serv Res 2008;8:179.
(16) Clayton M, et al. Advice given to patients about return to work and driving following surgery. Occup Med 2007;57:488-491.
(17) Clayton M, et al. A retrospective study of return to work following surgery. Occup Med 2007;57:525-531.
(18) Dawes HA, et al. Specialist nurse supported discharge in gynaecology: a randomised comparison and economic evaluation. Eur J Obstet Gynecol Reprod Biol 2007;130:262-270.
(19) de Vroome EMM, et al. Werkgebonden risicofactoren van ziekteverzuim en huisarts- en specialistenbezoek. Nederlands Tijdschrift Geneeskunde 2003;(81:127).
(20) Department of Health. Choosing health: making healthier choices easier. London; 2004.
(21) Dirksz AA, … , Brolmann HA. Werkhervatting na gynaecologische ingrepen; Pilot Study 2007.
(22) Dolan P. Modeling valuations for EuroQol health states. Med Care 1997;35:1095-1108.
(23) Franche RL, et al. Readiness for return to work following injury or illness: conceptualizing the interpersonal impact of health care, workplace, and insurance factors. J Occup Rehabil 2002 ;12:233-256.
(24) Gezondheidsraad. Beoordelen, behandelen begeleiden. Medisch handelen bij ziekteverzuim en arbeidsongeschiktheid Den Haag. 2005 Jul.
(25) Johansen P, et al. Analysis of need for sick leave after hysterectomy. Ugeskr Laeger 2008 Apr 21;170(17):1465-1468.
(26) Kikuchi I, et al. Questionnaire analysis of recovery of activities of daily living after laparoscopic surgery. J Minim Invasive Gynecol 2008 Jan;15(1):16-19.
(27) Kluivers KB, …, Mol BWJ, Brölmann HAM. Clinimetric properties of 3 instruments measuring postoperative recovery in a gynecologic surgical population. Surgery 2008 Jul;144(1):12-21.
(28) Kremer AM., Anema JR. Kwantificering van hersteltijden. Een gezamenlijk referentiekader voor bedrijfsarts en specialist. 2004 Jan.
(29) Loisel P, et al. A population-based, randomized clinical trial on back pain management. Spine 1997 Dec

15;22(24):2911-2918.
(30) Loisel P, et al. Disability prevention - New paradigm for the management of occupational back pain. 9[7], 351-360. 2001. Disease Management & Health Outcomes.
(31) Moller C, et al. Fast track hysterectomy. Eur J Obstet Gynecol Reprod Biol 2001 Sep;98(1):18-22.
(32) National Institute for Occupational Safety and Health. National occupational research agenda (NORA). Cincinnati: OH: US Department of health and Human Services. 1996.
(33) Oostenbrink JB, et al. Manual for cost studies, methods and standard cost-prices for economics evaluation in health care Board of health care insurance; 2004.
(34) Ottesen M, et al. Substantial variability in postoperative treatment, and convalescence recommendations following vaginal repair. A nationwide questionnaire study. Acta Obstet Gynecol Scand 2001;80:1062-1068.
(35) Ottesen M, et al. Short convalescence after vaginal prolapse surgery. Acta Obstet Gynecol Scand 2003;82:359-366.
(36) Robertson GS, et al. How long do patients convalescence after inguinal herniorrhaphy? Current principles and practice. Ann R Coll Surg Engl 1993 Jan;75(1):30-33.
(37) Samoocha D, ... Anema JR et al. Effectiveness of web-based interventions on patient empowerment: a systematic review and meta-analysis, Journal of Medical Internet Research (JMIR)(in press).
(38) Scharfenberg M, et al. "Fast-track" rehabilitation after colonic surgery in elderly patients--is it feasible? Int J Colorectal Dis 2007;22:1469-1474.
(39) Steenstra IA, et al. Prognostic factors for duration of sick leave in patients sick listed with acute low back pain: a systematic review of the literature. Occup Environ Med 2005 ;62:851-860.
(40) Stewart MA. Effective physician-patient communication and health outcomes: a review. CMAJ 1995 May 1;152(9):1423-1433.
(41) Stichting Informatie Gezondheidszorg. Landelijke Medische Registratie LMR (Prismant) Verrichtingen. 2006.
(42) van Amstel RJ, Anema JR, et al. Limited change in the quality of the social medical guidance and in the satisfaction of sick-listed patients, after collaborative projects between general practitioners and occupational physicians. Ned Tijdschr Geneeskd 2005;149:2407-2412.
(43) van Oostrom SH, …, van Mechelen W, Anema JR. Workplace interventions for preventing work disability. Cochrane Database Syst Rev 2009;(2)
(44) van Oostrom SH, Anema JR, …, van Mechelen W. Development of a workplace intervention for sick-listed employees with stress-related mental disorders: Intervention Mapping as a useful tool. BMC Health Serv Res 2007;7:127.
(45) Verbeek JH, et al. Early occupational health management of patients with back pain: a randomized controlled trial. Spine 2002 Sep 1;27:1844-1851.
(46) Waddell G, Burton AK. Is work good for your health and well-being? The Stationary Office 2006.
(47) Weevers HJ, ..., Anema JR, …, van Mechelen W. Work-related disease in general practice: a systematic review. Fam Pract 2005 Apr;22(2):197-204.
(48) WHO. International Classification of Functioning, Disability and Health. 2001.
(49) Young AE, …, Anema JR, et al. Return-to-work outcomes following work disability: stakeholder motivations, interests and concerns. J Occup Rehabil 2005 ;15:543-556

Financiële gegevens / Financial data

ZonMw budget

	Jaar / Year								
**Kostenpost**	*1*	*2*	*3*	*4*	*5*	*6*	*7*	*8*	**Totaal / Total**
Personeel	85,273	127,237	116,137	77,654	0	0	0	0	406,301
Materieel	10,000	3,000	2,000	0	0	0	0	0	15,000
Implementatie	0	0	0	0	0	0	0	0	0
Apparatuur	800	600	400	200	0	0	0	0	2,000
Overig	2,500	2,500	2,500	8,500	0	0	0	0	16,000
**Totaal / Total**	**98,573**	**133,337**	**121,037**	**86,354**	**0**	**0**	**0**	**0**	**439,301**

Co-financiering / Cofinancing

Naam co-financier / Name of cofinancier	Bedrag / Amount	Status

Bijzondere gegevens / Additional information

Vergunningen / Permits

	Verklaring nodig / Statement required?		Verklaring verkregen / Statement obtained?		
	Ja / Yes	Nee / No	Ja / Yes	Nee / No	Nog niet aangevraagd / Not applied yet
METC	X			X	
DEC		X		X	
WBO		X		X	

Onderschrijvingen / Assents

	Ja / Yes	Nee / No	N.v.t. / N.A.
Code biosecurity / Code Biosecurity		X	
Code openheid dierproeven / Code Transparency of Animal Testing		X	

Andere vergunningen / Other permits

Historie subsidieaanvraag / History grant application

Deze aanvraag is eerder ingediend bij het programma / This grant application has previously been submitted to the ZonMw programme:

DoelmatigheidsOnderzoek 2010-2012: deelprogramma Vroege Evaluatie van Medische Innovatie (VEMI)

Projectnummer / Project number:

80-82305-97-10050

Deze aanvraag is ook ingediend bij organisatie / This grant application has also been submitted to organization:

Ondertekening / Signatures

Naam projectleider en penvoerder:<br>J.A.F Huirne	Naam bestuurlijk verantwoordelijke:<br>T.J.F. Savelkoul
Plaats en datum:	Plaats en datum:
Handtekening:	Handtekening:
