# Supplementary material for: The Cost Effectiveness of a Tailored, Web-Based Care Program to Enhance Postoperative Recovery in Gynecologic Patients in Comparison With Usual Care: Protocol of a Stepped Wedge Cluster Randomized Controlled Trial
Source: JMIR Res Protoc. 2014 Jun 18;3(2):e30. doi: 10.2196/resprot.3236 (PMC4090379; doi:10.2196/resprot.3236)
Supplement: Supplementary file 4 [file resprot_v3i2e30_app4.pdf]

The Netherlands Organisation for
Health Research and Development

Laan van Nieuw Oost-Indië 334
2593 CE Den Haag
Postbus 93245
2509 AE Den Haag
Phone +31 (0)70 349 51 11
Fax +31 (0)70 349 51 00
www.zonmw.nl
info@zonmw.nl

Filenumber
171102015
Our reference
2014/00856/ZONMW

Date
January 21, 2014

Contact
Elisa Fung
Phone +31 (0)70 349 52 60
fung@zonmw.nl

To whom it may concern

Onderwerp
Confirmation of grant approval

Dear sir, madam,

I am writing to confirm that ZonMw has decided to approve a grant of € 439.301,- for the project entitled "*The cost-effectiveness of a transmural peri-operative care program for gynaecology*" This decision was communicated in a letter of October 20th 2010 (2010/29945/ZONMW) and is based on the positive recommendation of the committee of the "Health care efficiency research" programme.

Assessment
ZonMw prioritizes project proposal submissions based on scientific quality and relevance to the programme. The committee based its assessment on the quality of the project proposal, the reviewers' assessments and the project manager's response to the latter.

If this letter gives rise to any questions, please contact the individual listed in the letterhead.

Yours faithfully,

Marleen Jonker
Programme officer

ZonMw's main commissioning bodies are the Ministry of Health, Welfare and Sport (VWS) and the Netherlands Organisation for Scientific Research (NWO)
